# Revisiting microgenderome: detecting and cataloguing sexually unique and enriched species in human microbiomes

**DOI:** 10.1186/s12915-024-02025-6

**Published:** 2024-12-05

**Authors:** Zhanshan Sam Ma

**Affiliations:** 1 Computational Biology and Medical Ecology Lab, Kunming Institute of Zoology, Chinese Academy of Sciences, Kunming, China; 2 Department of Entomology, College of Plant Protection, Hebei Agricultural University, Baoding, China; 3 Microbiome Medicine and Advanced AI Lab, Cambridge, MA 02138 USA; 4 Faculty of Arts and Science, Harvard University, Cambridge, MA 02138 USA

**Keywords:** Microgenderome, Species specificity and specificity diversity (SSD) framework, Sexually unique species (US), Sexually enriched species (ES), Species specificity (SS), Specificity diversity (SD), Permutation test

## Abstract

**Background:**

Microgenderome or arguably more accurately microsexome refers to studies on sexual dimorphism of human microbiomes aimed at investigating bidirectional interactions between human microbiomes, sex hormones, and immune systems. It is important because of its implications to disease susceptibility and therapy, in which men and women demonstrate divergence in many diseases especially autoimmune diseases. In a previous report [1], we presented analyses of several key ecological aspects of microgenderome by leveraging the large datasets of the HMP (human microbiome project) but failed to offer species-level composition differences such as sexually unique species (US) and enriched species (ES). Existing approaches, for such tasks, including differential species relative abundance analysis and differential network analysis, possess certain limitations given that virtually all rely on species abundance alone or are univariate, while ignoring species distribution information across samples. Obviously, it is both species abundance and distribution that shape/drive the structure and dynamics of human microbiomes, and both should be equally responsible for the universal heterogeneity of microbiomes including the sexual dimorphism.

**Results:**

Here, we fill the gap by taking advantages of a recently developed computational algorithm, species specificity, and specificity diversity (SSD) framework (refer to the companion article) to reanalyze the HMP and complementary seminovaginal microbiome datasets. The SSD framework can randomly search and catalogue the sexually specific unique/enriched species with statistical rigor, guided by species specificity (a synthetic metric of abundance and distribution) and specificity diversity (SD). The SSD framework reveals that men seem to have more unique species than women in their gut and reproductive system microbiomes, but women seem to have more unique species than men in the airway, oral, and skin microbiomes, which is likely due to sexual dimorphism in the hormone and immune systems. We further investigate co-dependency and heterogeneity of those sexually unique/enriched species across 15 body sites, with core/periphery network analyses.

**Conclusions:**

This study not only produced sexually unique/enriched species in the human microbiomes and analyzed their codependency and heterogeneity but also further validated the robustness of the SSD framework presented in the companion article, by performing all negative control tests based on the HMP gut microbiome samples.

**Supplementary Information:**

The online version contains supplementary material available at 10.1186/s12915-024-02025-6.

## Background

Microgenderome or sexual dimorphism in human microbiome is an active research field that aims to investigate the bidirectional interactions between microbiota, sex hormones, and immune systems as well as their implications to disease susceptibility and therapy [1–9]. For example, gut microbiome is involved in the excretion and circulation process of sex hormones, most notably estrogen and androgens, and therefore influences the metabolism of estrogen and androgens. The sex hormones, in turn, may also influence the gut microbiomes. Specifically, beta-glucuronidase of the gut microbiome can convert conjugated estrogens to their deconjugated forms (as active deconjugated estrogen), which enters the enterohepatic circulation and act on estrogen receptor α and estrogen receptor β in the reproductive organs, muscle, nervous system, and vasculature. This mechanism is also true to non-ovarian estrogen in men and postmenopausal women, but not to ovarian estrogen in pre-menopausal women [9, 10].

According to Vermuri et al. [6], sexual dimorphism in immunity is mainly shaped by sex hormones and sex-linked immune response genes, and the GIT (gastrointestinal tract) is considered as one of the largest immune organs in human body and contains multiple immune cells that have profound influences on local and systemic inflammation. Gut microbiome drives diverse interactions locally with immune cells, which regulate the homeostatic environment and systemically in diverse tissues. Atop on the previous interaction chain is the sexual dimorphism or microgenderome (or microsexome), which consequently may lead to sex-dependent changes in local GIT inflammation, systemic immunity, and susceptibility to a range of inflammatory diseases. Furthermore, microgenderome may play a critical role in the response to the alternative therapies (such as probiotics, synbiotics, and fecal microbial transplant) designed to manipulate the gut microbiome. Those alternative treatments hold potential in the treatment of both psychological (e.g., anxiety and depression) and physiological diseases (such as gout and IBD) with different susceptibilities for men and women [6].

A foundation step in studying microgenderome is to dissect the sex difference in microbiota (microbial communities) including both community-level and species-level characteristics. In a previous study, Ma and Li [1] investigated seven community-level characteristics of microgenderome by leveraging the datasets from the NIH-HMP (human microbiome project) [11, 12]. Nevertheless, their study was virtually exclusively focused on aspects of microbial community ecology and networks, but microbial species-level insights were largely missing in that study [1]. This, of course, does not imply that species-level understanding of microgenderome is less important. For example, Simpson et al. [13] systematic review of 26 case studies on the relationship between gut microbes and anxiety disorders and depression reviewed conflicting evidence in terms of alpha and beta diversity. However, it was found that the disorders were associated with a higher abundance of proinflammatory species (e.g., *Enterobacteriaceae* and *Desulfovibrio*) and lower short-chain fatty acid producing-bacteria (e.g., *Faecalibacterium*). They suggested that several taxa and their action mechanisms may be associated with anxiety and depression pathophysiology via communication of peripheral inflammation to the brain. However, their review did not implicate methodological approaches to identify those interesting taxa. We further postulate that sex dimorphism such as different levels of enrichment of those special taxa could play a role in the different susceptibility of men and women to anxiety and depression disorders. In the present study, we fill the species-level gap in microgenderome research by identifying species that are unique or enriched, both statistically significant, in either the microbiome of male or female.

Investigating species-level sexual dimorphism is just as, if not more, important as holistic studies on the microbiota-level characteristics. This is not only because species (strictly, their populations) constitute microbiota but also because not all species are “born” equal in a microbiota, both functionally and compositionally. Compositionally, why some species are missing in women and why some others are missing in men are not only puzzling evolutionarily but also hinting certain biomedical signals important for understanding sexual dimorphism in disease susceptibility and/or therapy responses. Obviously, identifying microbial species that are unique or enriched in men or women is the very first step in solving the puzzle or gaining the biomedical insights.

Existing computational approaches for differentiating microbiomes at species or OTU levels can be grouped into three categories. The first category is the differential network analysis (DNA) originated from the studies on gene co-expression networks [14–16]. The second category is the so-termed differential species relative abundance (DSA) analysis and is the focus of recent methodological explorations in the field (e.g., [17–22]). The third category is the integration of machine learning with the previous DNA and/or DSA approaches, which is a new trend but still lacks systematic methods (e.g., [23–26]). Nearly all these existing approaches rely on species abundance alone and can be considered as univariate. In the companion article [27], both species abundance and distribution information are harnessed in order to differentiate the microbiome treatments at both species and community levels.

Back to the problem of identifying unique or enriched species in cohorts of microbiome samples, Ma [27] developed the so-termed species specificity and specificity diversity (SSD) framework that provides a feasible solution to the problem. The framework synthesizes both species abundance and distribution information across metacommunity, which captures critical aspects of community (metacommunity) heterogeneity [28]. The SSD framework consists of three components: first, the species specificity (SS) [29] measures the position of a species in the so-called specialist-generalist continuum, and it is attached to each species in the microbiomes of populations (e.g., male or female cohorts). Second, the specificity diversity (SD) is a new concept defined to measure the information (uncertainty) of SS with Renyi’s entropy in the form of so-termed Hill numbers [27], similar to the measure of biodiversity with Hill numbers [30]. However, different from biodiversity measures with Hill numbers, which measures the information (uncertainty) in local species abundance only, the SD measures the diversity of specificity that is a product of both local species prevalence and abundance share out of global populations. In other words, compared with traditional species diversity defined for species abundance, the new SD measures the information of both abundance and distribution synthesized by SS. This makes SS and SD possessing both local (e.g., male or female) and global (across sexes) information of individual species and assemblages of species, respectively. Third, a pair of stochastic permutation algorithms, termed specificity permutation (SP) and specificity-diversity permutation (SDP) tests were designed, with statistical rigor measured in *P*-value, to identify unique species (US) or enriched species (ES) in either men or women exclusively by leveraging the stochastic information (entropy) from SS and SD. Rather than resorting to deterministic “brute-force” one-by-one comparison, which can find exact solutions for a very small number of samples, the SSD framework produces solutions that are correct with statistical rigor (*P* < 0.05), that is, classifying the species status in error is a small probability event (*P* < 0.05).

Besides cataloguing unique (enriched) species in men or women as introduced previously, with species specificity and complex network approaches, we further reconstruct species specificity network (SSN) to gain insights on the co-dependence between species across all 15 microbiome sites of human body and to compare the holistic differences between men and women. In addition, we also reconstruct site heterogeneity network (SHN) to gain insights on the heterogeneity of within-body microbiome sites (e.g., skin vs. gut) in terms of species specificity, which can be considered as a comprehensive property of microbial biogeography.

As a side note, although the majority of existing literatures on microgenderome have been focused on gut microbiomes, the sexual dimorphism is not limited to gut microbiomes, and the sex gap is well known in respiratory diseases including cystic fibrosis (CF) and chronic obstructive pulmonary disease (COPD) [8]. While the sexual differences in prevalence, severity, and prognosis of CF and COPD are well documented, the pathophysiology of the sex differences is poorly understood. The lung microbiome is related to host immune response and health consequences and is important for understanding the sex differences found in respiratory diseases. An alternative path is to consider gut-lung axis, given that the gut microbiome plays a critical role in distant immune modulation in respiratory diseases. Both perspectives, i.e., the direct modulation of lung diseases by lung microbiomes and indirect modulation of lung diseases through gut-lung axis, are important avenues for studying pathophysiology of lung diseases, that is both the local (lung) and distant (gut) contributions to the pathophysiology of lung diseases are crucial for understanding the implications of microgenderome to lung diseases. Such cross-site perspectives should also be applicable to other interactions including gut-skin or gut-vaginal axis. Actually, our community-level approaches to microgenderome [1] were not limited to gut microbiome, and, instead, the HMP (human microbiome project) datasets we reanalyzed covered all major human microbiome sites including oral, skin, airway, gut, and vaginal samples.

## Results

### Species specificity lists of 15 human microbiome sites

**Fig. 1:**
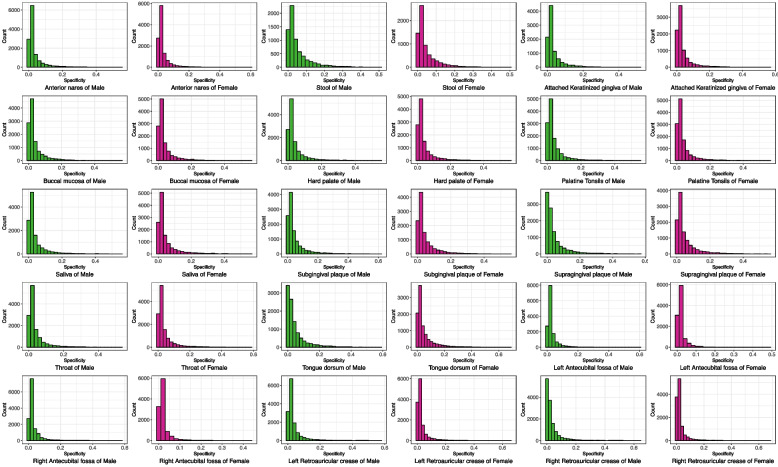
The histograms of species specificity (SS) for both sexes at each of the 15 body sites, computed from the HMP datasets: the green was for the male and the purple for the female

Table S1A-S1O in the online supplementary information (OSI) included 15 MS®-Excel sheets, each of which exhibited a pair of species specificity (SS) lists for the male and female at each of the 15 body sites. These 15 pairs of specificity lists are the input data for the following two permutation tests aimed to identify sexually unique, enriched species (US, ES) lists in each sex. Figure 1 illustrated 15 pairs (pair of the same site, e.g., saliva, of male and female) of specificity histograms for each of 15 body sites, to compare the male and female in their species specificity distribution (frequency). Both sexes appear to exhibit similar patterns in their histograms, but their difference or dissimilarity is not obvious in those histograms, and instead, the difference requires other kinds of analyses to reveal as exposed below. One apparent finding from the histograms in Fig. 1 is that the specificity distributions of both the male and female follow highly skewed, long-tail distribution, rather than symmetrical Gaussian distribution. This is similar to species abundance distribution, and it reminds of us that statistical moments such as mean and variance may not be effective parameters to characterize the species specificity. Instead, entropy can be more effective in summarizing the information contained in specificity values, as demonstrated by the new concept of specificity diversity [27].

### Specificity permutation (SP) tests for detecting sexually unique or enriched species

While the previous step of computing the SS lists from the OTU tables of the HMP datasets occupied the first two blocks in Fig. 8 (the flowchart of computational procedures of the SSD framework), the middle block produced the main results of this study by performing two permutation tests. The specificity permutation (SP) tests classify all microbial species at each body site into four categories (groups): unique species (US) in male, USs in female, enriched species (ES) in male, ES in female, plus the species without significant differences in SS between the male and female, termed insignificantly different species (IDS). The first four categories are further divisions of the significantly different species (DS) in SS between the male and female, i.e., the complement set of the IDS category. For the IDS category, we do not pursue their classification further given their lack of differences in SS between the male and female.

**Fig. 2:**
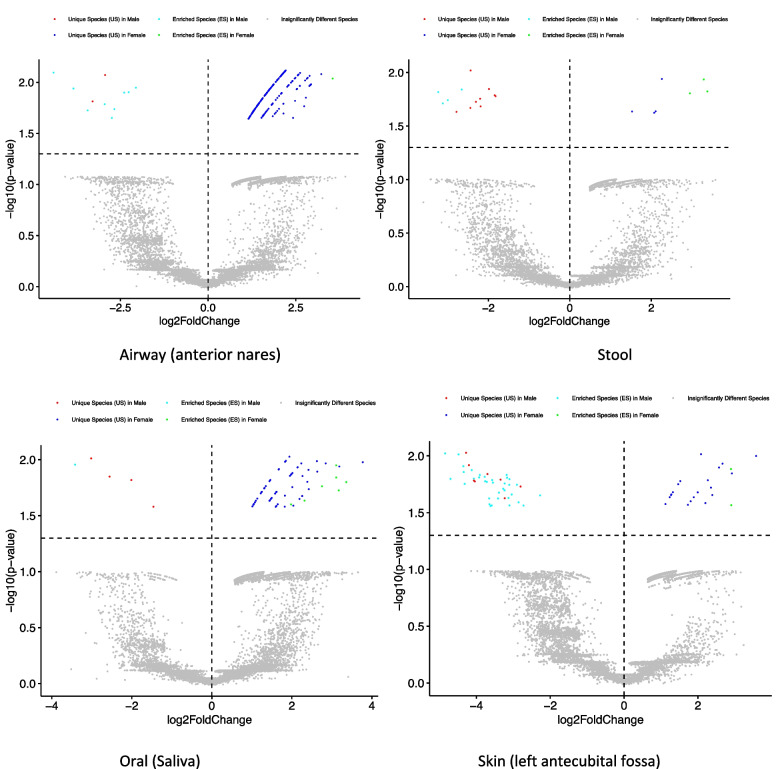
The volcano map showing the distribution of unique species (US), enriched species (ES), and the insignificantly different species, from specificity permutation (SP) tests: the *X*-axis represents the log-transformation of the specificity fold change between male and female, where fold change = *S*(male)/*S*(female) (*S* represents specificity of species); *Y*-axis represents the negative log-transformation of the *P*-value from SP tests of the specificity differences between male and female cohorts. The vertical dotted line at *X* = 0 represents fold change = 1 [i.e., *S*(male) = *S*(female)]; the points in the right side of this dotted line represent species with *S*(male)/*S*(female) > 1 [i.e., *S*(male) > *S*(female)]; the left points represent species with *S*(male)/*S*(female) < 1 [i.e., *S*(male) < *S*(female)]. The horizontal dotted line represents *P*-value = 0.05 [–log10 × (0.05) = 1.301]; the points above the line represent species specificity with significant differences between male and female, and the points below represent species of non-significant differences in specificity. Therefore, the grey points represent species of non-significant differences in species specificity between male and female, cyan points represent significant enriched species in male, green points represent significant enriched species in female, and red and blue points represent unique species in male and female, respectively. See Fig. S1A in the OSI for the volcanos of other sites; Fig. S1B also included the “directly drawn” [without using log(fold changes)] volcano graphs of these graphs

Table S2A-2O displayed the 15 sets of species categories (2 US, 2 ES) from the SP tests for the 15 body sites, one set per site. Figure 2, using four body sites (anterior nares, stool, saliva, and left antecubital fossa) as examples, displayed the distributions of the species categories in the form of standard “volcano” graphs, in which points representing the previously described five different species groups (2 USs, 2 ESs, and IDS) are scattered in a Cartesian coordinate plane (see the figure legend for details). The advantage of the volcano graph is its visualization of all species compared between the male and female (for each body site), plus the position of points and their distances signal the difference among species in terms of their specificity differences. Nevertheless, it is usually hardly possible to display species identities in limited page space, and therefore, the information in Table S2 is indispensable to identify species in each category. In addition, the volcano graphs of Fig. 2 adopted some adaptations from a standard volcano graph to circumvent an issue with log(fold change), which can be zero with microbiome datasets but is not an issue in genomics where volcano graphs are originated. In the companion article [27], the version of volcano graph directly drawn with specificity without using log(fold change) is illustrated. Figure S1 in the OSI presented the corresponding graphs without using adaptions (Fig. S1B) as well as the volcano graphs of other microbiome sites (Fig. S1A).

### Specificity-diversity permutation (SDP) tests for sexually holistic differences

While the previous section presented the SP tests for classifying the microbial species into unique and enriched ones, in this section, we present the results of SDP tests, which were aimed to test the holistic differences between two sexes in their respective microbial species groups, e.g., the “ES in male” vs. “ES in female” for comparison of their specificity diversity (SD) profiles. The SD profile refers to the SD values at a series of diversity order (*q* = 0, 1, 2, 3, 4), which collectively determines the species specificity distribution, similar with statistical moments for summarizing statistical distributions. Table S3A–S3F included 6 sub-tables corresponding to the SDP test results for the respective groups of “US in the male,” “US in the female,” “ES in the male,” “ES in the female,” “All significantly different species (DS),” and “All species (DS + IDS).”

The SDP tests for the first five species categories (of 2 USs, 2 ESs and DS) (*see* Table S3A-S3E) showed a consistent pattern, that is, the specificity diversity (SD) is different between the male and female in virtually all 15 body sites where the human microbiomes were sampled in the HMP project, across all diversity orders (*q* = 1–4) (the results for *q* = 0 are discussed below separately). The near 100% differences across all sites, orders, and, most conspicuously, all categories except for the last category of “all species” (DS + IDS) highlighted the significant differences between the male and female. The remarkable differences (nearly 100%) between both sexes in terms of the SD, except for the whole microbiome (all species) level, surpassed all of the differences previously measured with conventional ecological metrics such as species diversity, heterogeneity, and stochasticity, most of which exhibited less than 50% differences between both sexes (Ma & Li 2019). This also reflects the exceptionally high sensitivity of specificity diversity in detecting the microbiome differences.

The SD in Hill numbers at different diversity orders (*q*) are weighted differently by species specificity: at *q* = 0, specificity is weightless (explained further below); *q* = 1, the SD is weighed in proportional to species relative specificity, *q* > 1, weighted in favor of high specificity species. The SD (*q*) in Hill numbers can be considered as “the number of equivalent species” dictated by the weighing scheme of diversity order *q*. For example, when *q* = 0, the SD (0) in Hill number defaults to species richness (i.e., the number of species within the species group or assemblage). When *q* = 1, the SD (1) is the number of species with typical specificity level. When *q* = 2, the SD (2) is the number of species with dominant (higher) specificity level. The SD (3) and SD (4) are the numbers of species with even more dominance specificity levels.

**Fig. 3:**
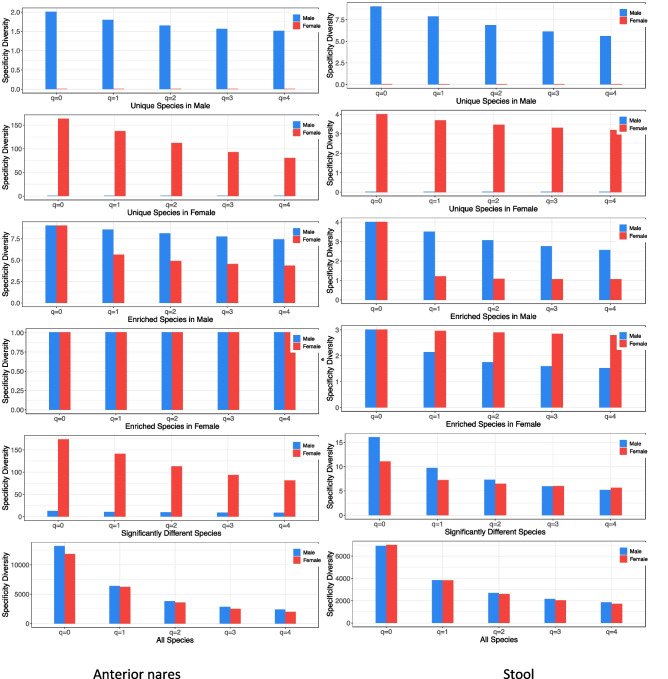
The specificity diversity (SD) for the “Anterior nares” and “Stool” body site: *Y*-axis shows the SD at different diversity order (*q* = 0–4, *X*-axis) for each of the six species categories (from top to bottom plot) including unique species (US) in male, US in female, enriched species (ES) in male, ES in female, species with significant differences, and all species (with + without differences). See Fig. S2 in the OSI for the results of SDP tests of other body sites

Figure 3 shows the SDs of different species categories based on the results presented in Table S3A-3F, using two body sites (anterior nares and stool) as examples.

### Specificity-diversity permutation (SDP) tests for the SD(q = 0) or species richness

**Fig. 4:**
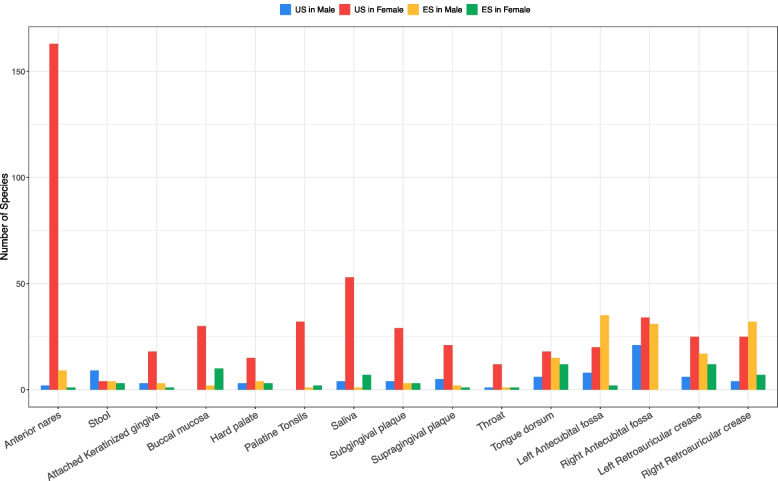
The numbers of unique species (US) and enriched species (ES) at each body site: the number of the US in the female is approximately 6–16 times more than the number of the US in the male, while the number of ES is similar in both sexes

In the previous section, we introduced the SDP results for the diversity orders of *q* > 1, and here we focus on the SD in Hill numbers at *q* = 0. The SD (*q* = 0) or SD (0) defaults to species richness of a species group (assemblage) and, it is the same *species richness* concept as in traditional biodiversity research and community ecology. From this perspective, SD (0) or species richness could be less interesting for specificity research since the specificity does not weigh in the computation of SD (0) at all. However, the SD(0), when computed for US/ES/DS/IDS, still makes important sense. For example, one would intuitively expect that the numbers of US in the male and female should be roughly equal. As shown in Table S3 and further summarized in Table S4 and Fig. 4, the numbers of the USs in the female across all body sites but stool are nearly *7* times (0.5 to 30) of those in the male on average (Fig. 4). The stool seems to be an exception with 9 US in the male and 4 US in the female. In particular, the biggest difference occurred in the airway (anterior nares: 2 US in the male and 163 in the female) and oral microbiomes (e.g., buccal mucosa: with 0 in the male and 30 US in the female); smallest occurred in the stool (actually more US in the male as mentioned previously). The differences between both sexes seem to be modest in the case of skin microbiomes.

A general pattern exhibited by Fig. 4 and Table S4 regarding the differences in the US numbers between the male and female is airway (anterior nares) > oral > skin > stool, with females having more US than males except for the stool, for which the male has more US number. In contrast, the difference in the number of ES between the male and female is 128 vs. 65 (Table S4), with the male having twice more ES than the female. Compared with the US species group, the difference is obviously much smaller and is similar to most ecological metrics compared in our previous study [1].

To verify that the previously exposed difference between the male and female is not a statistical artifact, we further redo the analysis with a different *P*-value of 0.01. Although both *P*-values of 0.05 and 0.01 made differences, the results (the whole Table S4) were the same after the adjustments with FDR control. Therefore, we believe that the differences between the male and female revealed in this study are not statistical artifacts.

### Species specificity network (SSN) and site heterogeneity network (SHN)

**Fig. 5:**
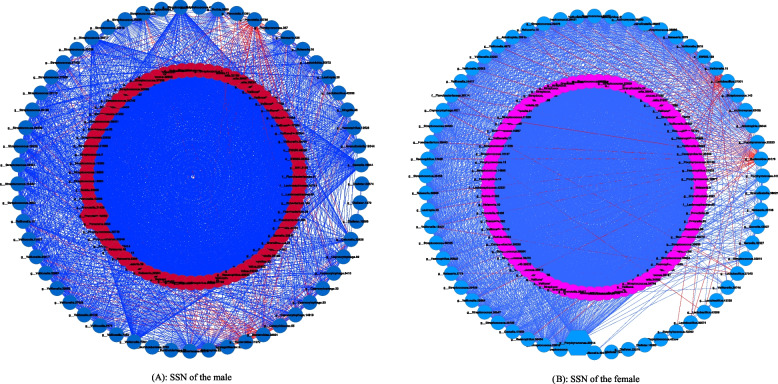
The species specificity network (SSN) of the male (**A**) and female (**B**): core nodes are in pink color, and periphery nodes are in cyan color; positive links are in green and negative links are in red; hexagon represents for hub. See Fig. S3 in the OSI for the top two strongest clusters detected from the SSNs, in which both male and female exhibited the same patterns: core and periphery forms the top two strongest clusters, and the within core links seem to be positively only

We constructed the SSNs for the male (Fig. 5A) and female (Fig. 5B) cohorts with the same computational procedures (computing Spearman’s correlation coefficients for the species that occurred in all 15 sites sampled by the HMP, using FDR control with *P*-value = 0.01) as routinely used for constructing ecological (species) correlation networks (e.g., [36]).

Among the fundamental network properties (metrics) we computed, all but one property exhibited rather close values, and the exceptional property was the *P/N* ratio (ratio of positive to negative links in network, *see* [1] for its formal definition) (male = 36.0 vs. female = 55.3), that is, the SSN of the male contained fewer negatively links than that of the female. However, topologically, both the networks have different hubs: the SSN of the male is *Prevotella* belonging to the core structure, and the SSN of the female is *Porphyromonas* belonging to the periphery structure. Overall, both the SSNs of both sexes are rather similar with each other (Fig. 5A, B).

Besides the high similarity in the basic network properties, the core/periphery structures in the SSNs for the male and female also exhibited remarkable homogeneity between the male and female. In both sexes, the core structures form the strongest clusters (Fig. S3A and Fig. S3B), and the periphery form the second strongest cluster (Fig. S3C and Fig. S3D). In addition, virtually all negative links are in the periphery, and the core seems to contain positive links only. All these findings are expected from the properties of core/periphery network structures: core consists of densely linked nodes, and periphery is sparsely connected to core and internally weakly and sparsely linked with each other. We postulate that the core/periphery structures dichotomy may offer hints for searching for the core of human microbiomes as pursued by the human microbiomes [11, 12], for which we seem to still lack a consensus.

**Fig. 6:**
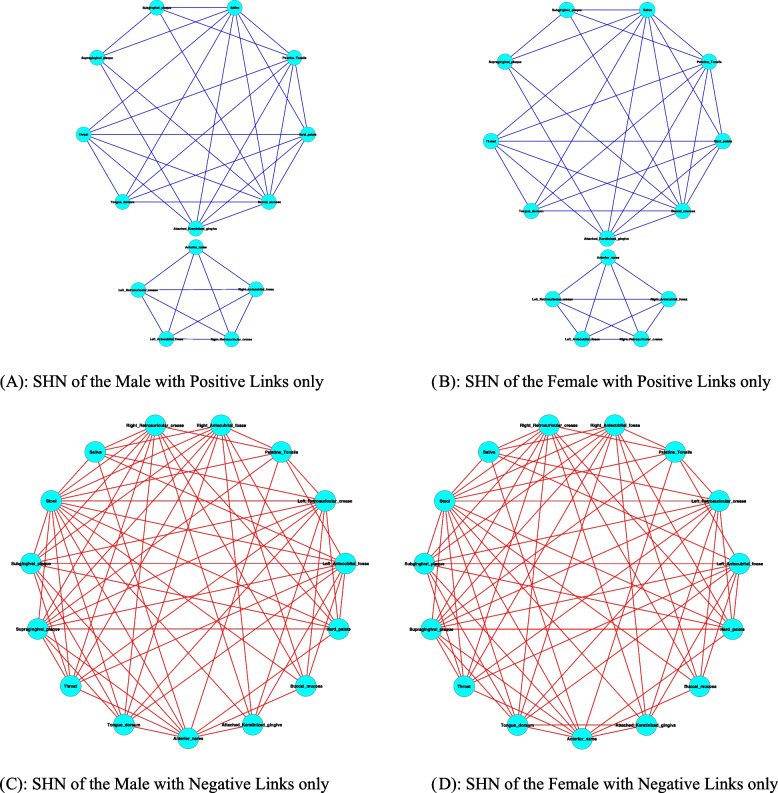
The site heterogeneity network (SHN) for the male (**A** and **C**) and female (**B** and **D**), respectively: to facilitate visual inspection, each SHN network was decomposed as two sub-networks of positive and negative links. Two observations can be made: (*i*) both sexes show the virtually same SHN structures; (*ii*) positive links exist within the sites of the same habitat except for the anterior nares, and negative links occurred between habitats (stool vs. oral, stool vs. skin, oral vs. skin,…). See Fig. S4 in the OSI for the total graphs (positive and negative networks restored as whole networks)

The male and female also displayed exceptional similarity in the site heterogeneity network (SHN) (Fig. 6). If we lump together the 15 sites as four habitats consisting of oral (9 sites), skin (4 sites), airway (1 site), and stool (1 site), then we see that the positive links only occurred within 9 oral site, within 4 skin sites and airway sites, but the negative links occurred only between the four habitats. This suggest that each of the four habitats (oral, skin, airway, and stool) are indeed distinctive as intuitively perceived; nonetheless, both sexes are rather homogenous at their respective sites, that is, both males and females possess the same or similar site heterogeneity in terms of species specificity co-dependency.

### Seminovaginal microbiome (SVM) analysis with SSD framework

In the previous section, all of the analyses and results are based on the HMP datasets, which are balanced cohorts with sufficiently large sample sizes (with approximately same sample sizes of > 100 +). Nevertheless, the HMP only sampled 15 male microbiome sites without sampling the male reproductive system microbiomes. Although three vaginal microbiomes (excluded from this reanalysis) were indeed sampled in the HMP, it is not possible to compare the reproductive system microbiomes based on HMP datasets. To fill this gap, we reanalyzed a seminovaginal microbiome dataset that sampled 23 couples before and after sexual intercourse with 69 samples originally reported by Mandar et al. (2015) who labeled the three treatments of samples as: CM = 23 semen samples (before sexual intercourse), CNA = 23 vaginal samples (before sexual intercourse), and CNB = 23 vaginal samples (after sexual intercourse). Given that the sample sizes of the seminovaginal microbiome (SVM) study was only 23 and is hardly comparable with the previous HMP datasets, we separately explain the SVM results here in the discussion.

We used the exactly same computational procedures previously used for analyzing the HMP datasets for comparing both sexes at each of the 15 body sites, to reanalyze the SVM datasets in pairwise manner: CM vs. CNA, CM vs. CNB, and CNA vs. CNB. The first two pairs compare the male vs. female, comparing semen samples (CM) either with vaginal microbiome before (CNA) or after (CNB) sexual intercourse. The last comparison (CNA vs. CNB) actually compared two vaginal samples before and after sexual intercourse, which is not the focus of this study.

**Fig. 7:**
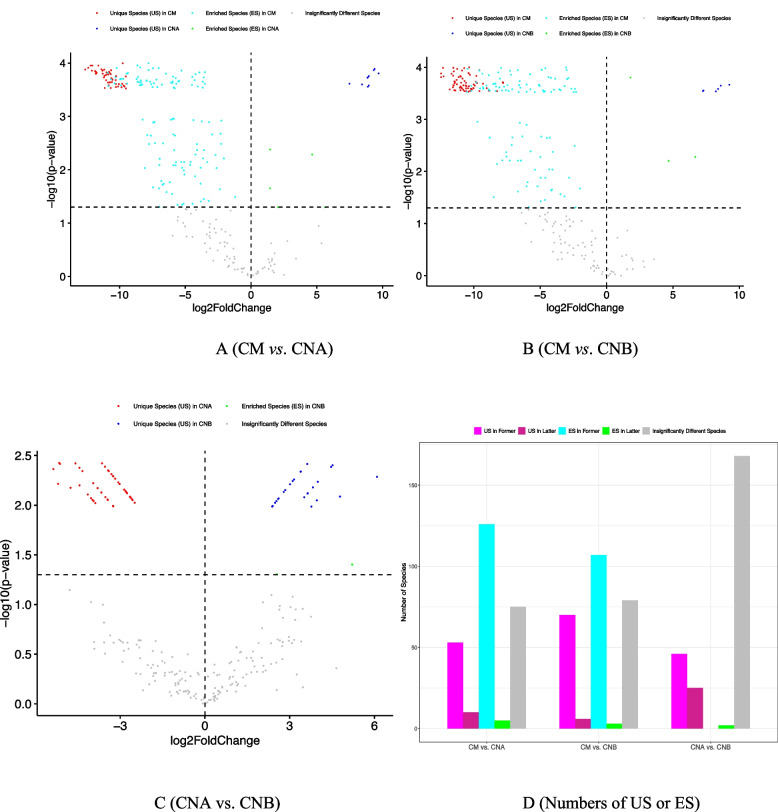
The comparisons of seminovaginal microbiomes (CM = semen, CNA = vaginal before sex, CNB = vaginal after sex): CMA vs. CNA, CM vs. CNB, and CAN vs. CNB. **A**–**C** are volcano graphs of US (unique species), ES (enriched species), and insignificantly different species, and (**D**) further illustrated the species numbers of different species categories. Similar to gut, men seem to have more US/ES species than women, and the sexual intercourse seems to raise the level of the differences. See Fig. S5-S6 for the other results (histograms, SDP tests) of the seminovaginal microbiome comparisons

Table S5 exhibited the species specificity lists of CM, CNA, and CNB, and Fig. S5 showed the histograms of those specificity distributions. Table S6 listed the species categories (US, ES) classified with SP tests from each of the three pairwise comparisons, and Fig. 7A–C here showed the three volcano graphs from the three pairwise comparisons. Figure 7D further summarized the comparisons of the US/ES/IDS numbers between the male and female reproductive system microbiomes (also refer to Table S8). It turned out that the semen (CM) microbiome has significantly larger US number than the vaginal microbiomes both before (CNA) and after (CNB) sexual intercourse, and the difference level actually increased from 5.3 (53/10) to 11.7 (70/6) times after sexual intercourse, that is, the sexual intercourse doubled the differences between the male and female reproductive systems, which is somewhat puzzling and we do not have an explanation.

During the peer review process, an anonymous expert reviewer suggested the following hypothesis to interpret the above-mentioned puzzle. Since only the vaginal microbiome is measured both before and after, the change observed lies there—it must mean that species shared with the seminal microbiome pre-intercourse no longer are shared with it post-intercourse because they have been lost in the vaginal microbiome following intercourse. This seems like the opposite of a transfer of microbiota, rather like the mechanical and immune process made the vaginal ecosystem hold less diversity for a while, which in turn raised the difference between men and women after sex intercourse. A further shared species analysis [40] also confirmed that the number of shared species numbers between man and woman decreased significantly after sex (down from 77 to 71%), equivalent to rising difference after sex intercourse.

Table S7A-S7F exhibited the results of specificity diversity permutation (SDP) tests for the seminovaginal microbiome (SVM) datasets. Overall, the specificity diversity (SD) between man and woman are significantly different both before and after sex, while the SD between two samples of woman is usually not significantly different except for the species assemblages of US. These results should be expected.

## Conclusions and discussion

Microbiomes are dynamic systems influenced by numerous internal and external (environmental) factors including sex, age, diet, physical activity, stress, temperature and humidity, medicines, disease, and bacteriophage, all of which may contribute to short- and long-term dynamics in microbiomes [41]. It is critical but often unknown whether the changes are only quantitative (only abundances) or qualitative (loss or gain of microbes) [41]. Our previous analysis of the HMP datasets with the SSD framework demonstrates a powerful approach to determine the *qualitative* difference between both sexes. Specifically, we detected and compiled the catalogues of US and ES species in either male or female at the species level and further measure the holistic difference, in terms of the specificity diversity (entropy of species specificity), between microbial assemblages (groups) or communities. We believe that those catalogues contain invaluable hints for investigating microgenderome such as sexual differences in certain disease susceptibilities.

Some authors argue that the term microsexome is a more appropriate term than microgenderome given that the former is a social selection (construct) and the latter is a biological selection (construct) [42]. Some researchers suggest that the effect of “gender” on the gut microbiome is very small compared with that of “sex.” However, both are obviously compounded, and it is hardly separable in some cases, for this, Kim [9] suggested the term “microsexome/genderome.” It is noted that the HMP datasets we reanalyzed were labeled with biological sexes as operationalized by the researchers.

A comparison between the results from the HMP and SVM studies suggests an interesting observation: the male vs. female differences in ES number in the reproductive system is consistent with that of gut microbiome, but opposite with those of oral, skin, and airway microbiomes. The relatively small sample sizes of the SVM datasets remind us that the comparisons/integrations of the findings from both HMP and SVM datasets should be cautioned. In summary, ignoring the caution on the sample size differences, in gut and reproductive microbiomes, men seem to have more US/ES than women, but women seem to have more US than men in airway, oral, and skin microbiomes. We do not have an explanation for the observation. During the peer review process, an anonymous review expert suggested that it could be that testosterone-driven skin metabolites feed some bacteria to bloom, reducing diversity and pushing other bacteria down below detection threshold—appearance of more unique skin taxa in women, similarly with the effect of estrogen versus testosterone on local immunity. This hypothesis certainly makes sense. As reviewed previously, existing literature certainly supports the notion that hormones, immune systems, and microbiomes are closely interwoven with each other in shaping the sexual dimorphism of microbiomes or the microgenderome.

Finally, we present a reliability (robustness) test for the SSD framework based on the HMP datasets per an insightful suggestion from the anonymous expert reviewer. The test scheme is to use all negative treatment (samples) as control to evaluate the reliability or effectiveness of the SSD framework, including the robustness of the previous findings on microgenderome. We choose the gut microbiome (stool samples) of the HMP datasets as example to discuss the test results here. The test design is actually quite simple. We compare the gut microbiome dataset of men with itself and the gut microbiome dataset of women with itself, respectively, as if an error of dataset misplacement (male/female swap) was committed in previous analyses in the section of results. We further designed two schemes to pre-prepare the datasets. One is to simply duplicate the dataset of male (or female) and then compare the two (duplicated) treatments, and another is to divide the dataset of male (or female) into two parts and then compare the two (divided) parts. With the two schemes for pre-preparing the treatments, the all negative control tests are performed for men and women gut microbiome, respectively, and a total of four sets of SSD framework analyses were conducted including (*i–ii*) men vs. men and women vs. women with duplicated treatment and (*iii–iv*) men vs. men and women vs. women with half-divided treatment. Table S9 [4 MS-Excel sheets S9A-S9D from the specificity permutation (SP) tests] and Table S10 [from the specificity diversity permutation (SDP) tests] exhibited the results from the four sets of SSD analyses.

Ideally, if the SSD framework performs perfectly, with the above-designed all negative controls, no species should exhibit significant differences in species specificity, and no US/ES species should be detected. The actual test findings with the HMP gut microbiome can be summarized as follows. (*i*) With the duplicated treatment scheme for the male gut microbiome, the SP test performed perfectly, no species was found with significant difference in species specificity (Table S9A). (*ii*) With the duplicated treatment scheme for the female gut microbiome, only a single species (f*_Ruminococcaceae*.617) was found enriched (Table S9B). (*iii*) With the half-divided treatment scheme for the male, five species were detected as with significant difference in species specificity including one unique species and four enriched species (Table S9C). (*iv*) With the half-divided treatment scheme for the female, eight species were detected as with significant difference in species specificity including three unique species and five enriched species (Table S9C).

The fact that only a single false positive species was found with the duplicated treatment scheme shows that the SSD framework is rather reliable. As to the finding that a few false positives were detected with the divided treatment scheme, it simply suggests that the sample sizes may have significantly influences on the power of the SSD framework, which should be expected. This is because, with the divided treatment scheme, the sample size of both treatments compared are reduced by half. In the case of the gut microbiome datasets from the HMP, there were 95 stool samples in both male and female treatments, respectively. With the duplicated treatment scheme, 95 samples were compared, which is the same as how the previous normal comparisons between men and women were performed. However, with the half-divided treatment scheme, 47 and 48 samples (total = 95) were compared, and the sample sizes of both “new” treatments compared were halved. Even with the half-divided treatments, the performance of SSD framework can still be considered as reliable given that only 8 (out of 8000 + species in gut microbiomes) false positive (type I errors) failed the species specificity tests.

Table S10 showed the SDP test results for all negative control tests, and it turned out that none of the SDP tests performed found any false positives under both duplicated and divided treatment schemes. In other words, the SDP tests performed perfectly as designed for the human gut microbiome datasets, which shows that the SDP tests are reliable even when the sample sizes were halved.

## Material and methods

### Human microbiome project (HMP) and seminovaginal microbiome datasets

The HMP datasets of US-NIH consists of 242 healthy adults with 129 male and 113 female individuals, each of whom was sampled at 15 (male) and 18 (female) body sites, respectively [11]. We excluded the three vaginal sites from this study since there were not samples from the male reproductive systems in the HMP study. The 16S-rRNA reads of V1-V3 regions from amplicon sequencing are used to obtain the OTU (operational taxonomic unit) tables at 97% similarity or species-level similarity. We actually used the OTU tables computed and released by the HMP project (https://www.hmpdacc.org/), at which both the tables and metadata information are available for public accesses.

To remedy a deficiency of the HMP in lacking comparative reproductive microbiomes, we reanalyzed the datasets of the 46 vaginal and 23 semen microbiome samples from 23 couples both before and after sexual intercourses, originally collected and published by [31]. The 69 samples (3 samples per couple, one semen, one vaginal before sexual intercourse, and another vaginal after sexual intercourse) were amplicon-sequenced to obtain 16 s-rRNA reads, which were further processed with the Mothur software pipeline to generate the OTU tables. In the present study, we used the OTU tables generously offered to us from the original investigators of Mandar et al. [31].

### The species specificity and specificity diversity (SSD) framework for identifying sexually unique or enriched species

As illustrated in Fig. 7, the SSD framework consists of two metrics, i.e., species specificity (SS) and specificity diversity (SD), as well as two corresponding statistical tests, i.e., specificity permutation (SP) test and specificity-diversity permutation (SDP) test. Refer to Ma [27] for the detailed information on the SSD framework, including the computational algorithms and implementation code.

Assume there is a pair of treatments (i.e., habitats in the terminology of SSD framework), and each represents the microbiome samples from either a male or female cohort exclusively. In the case of the HMP study, the male “habitat” supports 129 individual men and the female “habitat” supports 113 individual women. Each microbiome sample from each individual subject (either man or woman) represents a local microbial community, all samples from one single treatment (sex or habitat) constitute a metacommunity, and there are therefore two microbial metacommunities, one for male hosts and another for female hosts. The two metacommunities constitute the microbial landscape of human microbiome. In the case of HMP datasets, the landscape spans two habitat types, i.e., male and female, consisting of 129 and 113 local microbial communities, respectively.

We treat the 15 body sites from an individual as 15 different habitat types, and consequently, we have 15 sets of microbial landscapes as described previously. In other words, we compare the microbiomes of males and females for each of the 15 body sites, respectively. For example, we compare the stool samples of males and those of females by treating all male stool samples as one metacommunity and all female stool samples as another metacommunity, and both metacommunities constitute the microbial landscape of the gut (stool) microbiome. We have 15 sets of microbial landscapes, corresponding to 15 body sites sampled by the HMP.

The SP test is a statistical test based on the species specificity (SS) metric (concept) and principle of permutation (randomization) test [32], and it is designed to classify species into unique and enriched species for each treatment (male or female). The unique species (US) in male corresponds to missing species in female, and vice versa. Similarly, the enriched species (ES) in male corresponds to depleted species (DS) in female, and vice versa.

The species specificity (SS) concept (metric) is proposed by Mariadassou et al. [29] as a reincarnation of the traditional generalist-specialist paradigm extensively studied in macrobial ecology of plants and animals [33]. The values of SS range between 0 and 1, with *0* indicating that the species is absent in local habitat (*h*) and *1* indicating that the species is a perfect indicator for that (local) habitat—the species always exists and only exists in the local habitat (but nowhere else in the landscape of all habitats). Different from species abundance, which is univariate, SS is a synthetic metric of species abundance and species distribution in the form of species prevalence (presence/absence across samples).

**Fig. 8:**
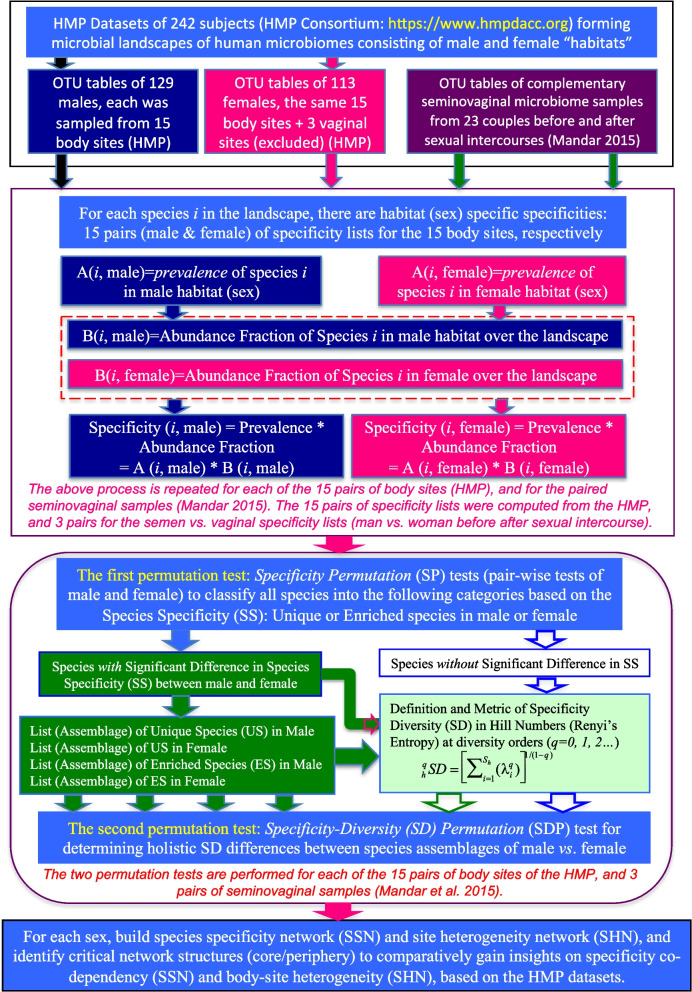
Diagram illustrating the computational procedures of species specificity and specificity diversity (SSD) framework: (i) the top block—the HMP datasets as input, (ii) the 2nd block—definition of species specificity (SS), (iii) the 3rd block—definition and algorithms of specificity diversity (SD) and specificity permutation (SP) and SD permutation (SDP) tests, (iv) the bottom block—network analysis for specificity co-dependency and human body site heterogeneity in specificity

As illustrated in Fig. 8, to define and compute the SS, one needs at least two habitat types, e.g., the male (M) and female (F) populations (habitats) or men vs. women. In this case, there are *h* = 1, 2 or, two habitat types, M and F. For each habitat type (M or F), there are *m* male (M) individuals and *n* female (F) individuals. Assume one microbiome sample is taken from each individual subject (in fact, more accurately, taken from one of the 15 sites of an individual, i.e., using one of the 15 body sites sampled by the HMP to make the example) of the (*m* + *n*) individuals. The previous setting is rather simple using traditional terminology of biomedical experiment design, we have two treatments (M and F), consisting of *m* and *n* microbial samples, respectively. However, to define the SS, here, we need to use the terminology of community ecology. Each individual microbiome sample is a local microbial community, and hence, there are *m* local communities in the M habitat (treatment) and *n* local communities in the F habitat. The *m* local communities in the M habitat constitute a male metacommunity (community of local communities), and *n* local communities in the F habitat constitute a female metacommunity. Both the metacommunities constitute the landscape of human microbiome. The point is that, in fact, the specificity is defined on the scale of landscape, and the implication is that the specificity of each species is directly influenced by those of other species in its metacommunity and is indirectly influenced by the species in the alternative metacommunity (hence, the whole landscape). This is obviously rather different from apparently similar species abundance, although both can be represented with a probability distribution, species specificity distribution (SSD), or species abundance distribution (SAD), which is familiar for ecologists and biostatisticians.

While species specificity is both species and habitat-specific, the specificity diversity (SD) defined by Ma [27] is an assemblage/community level metric. The SD synthesizes the specificity *information* or *uncertainty* (lack of information) carried by all species within an assemblage (or community), and it is defined as Renyi’s [34] entropy in the form of Hill numbers. The relationship between SS and SD is similar to that between species abundance (SA) and species diversity. Nevertheless, there is a fundamental difference that is due to the cross-habitat or landscape nature of species specificity as explained previously, namely, both SS and SD are influenced by species in alternative habitats, for example, the microbiomes of men vs. women.

With the pair of specificity and specificity diversity metric, SS and SD, Ma [27] designed corresponding pair of permutation algorithms, specificity permutation (SP) and specificity diversity permutation (SDP) tests. The SP test is developed to classify all species in a pair of treatments (e.g., male vs. female or the microbial landscape of human microbiomes) as the following four interesting categories (assemblages): unique species (US) in male, US in female, enriched species (ES) in male, and ES in female, plus the fifth category—statistically indifferent species in specificity. Actually, the SP test first classify all species in both males and females as two super-categories: the previous fifth category of “indifferent species” and “significantly different species”—all species with statistically significantly different specificity between male and female treatments. As expected, the four categories are the further divisions of the latter super-category. All of the classifications (US, ES, etc.) were made based on standard permutation tests with 1000 times of re-sampling with a default significance level (threshold) of *P*-value = 0.05, further adjusted with the FDR (false discovery rate) control.

The SDP test is designed to test the holistic difference in SD between the two assemblages of the *same* kind of species category from the two alternative habitats. We perform six SDP tests as illustrated in Fig. 8: four SDP tests for the 2 US and 2 ES categories, the fifth for “all significantly different species” (in SD), and the sixth for “all species” (without considering the SS). Note that in each of the six SDP tests, the “same species group (assemblage)” are tested for their SDs in the two mutually alternative habitats of both sexes. For example, for species the ES group, there are actually two specificity lists, one in the male treatment and another in the female treatment. This is because specificity is both species and habitat specific, and each species has a pair of specificity values, one in the male treatment (habitat) and another in the female treatment (habitat).

It should be emphasized that, as noted in Fig. 8, the above outlined computational process is actually performed independently for each of the 15 body sites sampled by the HMP, that is, we compare 15 pairs of the body sites of both sexes, respectively, e.g., comparing the gut microbiomes of male and female cohorts.

### Species specificity network (SSN) and site heterogeneity network (SHN)

By treating the 15 sites of the male as 15 different habitats, and we can compute a specificity list for all species from each habitat, and the resulting 15 specificity lists constitute a species specificity table (matrix) for the male. Similarly, we can obtain a species specificity table (matrix) for the female. Similar to the usage of species abundance table for constructing species abundance correlation (cooccurrence) network, we can build a species specificity correlation (codependency) network for each of the two specificity tables of the male and female, termed species specificity (co-dependency) networks (SSN), and then compare their network properties or structures.

Alternatively, from the same species specificity tables, we can build the site heterogeneity network (SHN) for each sex by computing the pair-wise correlation between microbiome sites in species specificity across all microbial species. Obviously, the specificity tables (matrices) used for building the SSN and SHN are transposed from each other, but both networks highlight different aspects of specificity co-dependency as measured by the correlation in species specificity. One obvious distinction is that the nodes of SSH are species, and the nodes of SHN are 15 body sites.

While the SSN highlights the specificity co-dependency (correlation) of species across 15 body sites, the SHN highlights the specificity correlation among 15 different body sites across all species in the specificity table. The SHN equivalently measures the similarity or heterogeneity between body sites (habitats) in microbial specificity. By comparing the SHN for male and female, we can gain insights on the similarity or difference between men and women in their specificity from site perspective. In particular, we inspect and compare the positive vs. negative correlation relationships among different body sites.

We used species that occurred in all 15 sites for the network analysis, i.e., species with specificity > 0 in all sites. Spearman’s correlation coefficients with FDR (false discovery rate) control (with *P*-value = 0.01) were computed and used to build the SSN and SHN; both are correlation networks built from mutually transposed specificity tables. We further detect the core/periphery structures (CPS) from the correlation networks. The CPS refers to the distinctions of network nodes as densely connected core vs. sparsely linked periphery (both within the periphery as well as with the core nodes). We use the same computational procedures and programs developed by Ma and Ellison [35, 36], which were developed for species dominance networks but are equally applicable to the network analysis of SSN and SHN in this study. Further information on CPS/HSS is referred to [37–39]. Finally, it should be noted that the network-building approaches specifically, Spearman’s correlation coefficients, we used in this study is likely not optimal. Our choice is mainly to keep consistent with the previous regular species cooccurrence network analysis used in microgenderome analysis [1]. Some alternative approaches such as SparCC algorithm [43] and power graph model [44] can be more effective in building the specificity networks, especially when the network is at very large scale [44].

## Supplementary Information

Supplementary Material 1.

Supplementary Material 2.

## Data Availability

All data generated or analyzed during this study are included in this published article, its supplementary information files, and publicly available repositories. The HMP (human microbiome project) datasets are available from the HMP data resources site: https://portal.hmpdacc.org/. The SVM (seminovaginal microbiome) datasets are available from the supplementary information of Mändar R, Punab M, Borovkova N, Lapp E, Kiiker R, Korrovits P, Metspalu A, Krjutškov K, Nolvak H, Preem JK, Oopkaup K. Complementary seminovaginal microbiome in couples. Research in Microbiology. 2015 Jun 1;166(5):440–7.

## Abbreviations

CPS: Core/periphery structure
DS: Depleted species
ES: Enriched species
HMP: Human microbiome project
OSI: Online supplementary information
OTU: Operational taxonomic unit
SAD: Species abundance distribution
SD: Specificity diversity
SDP: Specificity diversity-permutation
SHN: Site heterogeneity network
SP: Specificity permutation test
SS: Species specificity
SSD: Species specificity and specificity diversity framework
SSN: Species specificity networks
US: Unique species = exclusive species
