# Supplementary material for: Revisiting microgenderome: detecting and cataloguing sexually unique and enriched species in human microbiomes
Source: BMC Biol. 2024 Dec 5;22:284. doi: 10.1186/s12915-024-02025-6 (PMC11622641; doi:10.1186/s12915-024-02025-6)
Supplement: Supplementary file 2 — Supplementary Material 2. [file 12915_2024_2025_MOESM2_ESM.pdf]

Online Supplementary Information for Ma (2022) "Revisiting Microgenderome"

Table of Contents

Part I: Tables S3-S4 & Figures S1-S4 (*Computed from HMP Datasets*): Pages 1-28
Part II: Tables S7-S8 & Figures S5-S6 (Computed from Mandar *et al*. (2015) complementary seminovaginal microbiomes datasets) (Pages 29-34)
Part III: See separate MS-Excel File

Part I: Tables S3-S4 & Figures S1-S4 (*Computed from HMP Datasets*)

Table S3A. The specificity diversity (SD) permutation (SDP) tests for the species category of unique species (US) in the male between both sexes

Microbiome	Treatments	Order	Male	Female	Delta	Lower (95%)	Upper (95%)	*p*-Value
Anterior nares	Male vs. Female	***q=0***	2.000	0.000	2.000	-0.686	0.776	0.000
		q=1	1.786	0.000	1.786	-1.130	1.144	0.000
		q=2	1.642	0.000	1.642	-1.264	1.261	0.000
		q=3	1.555	0.000	1.555	-1.283	1.274	0.000
		q=4	1.503	0.000	1.503	-1.274	1.263	0.000
Stool	Male vs. Female	***q=0***	9.000	0.000	9.000	-4.248	4.200	0.000
		q=1	7.829	0.000	7.829	-6.896	6.729	0.000
		q=2	6.831	0.000	6.831	-7.307	7.133	0.003
		q=3	6.087	0.000	6.087	-7.191	7.031	0.057
		q=4	5.568	0.000	5.568	-6.998	6.852	0.103
Attached Keratinized gingiva	Male vs. Female	***q=0***	3.000	0.000	3.000	-0.628	0.686	0.000
		q=1	2.972	0.000	2.972	-0.921	1.029	0.000
		q=2	2.945	0.000	2.945	-1.055	1.168	0.000
		q=3	2.920	0.000	2.920	-1.094	1.206	0.000
		q=4	2.896	0.000	2.896	-1.104	1.213	0.000
Buccal mucosa	Male vs. Female	***q=0***	0.000	0.000	0.000	0.000	0.000	0.000
		q=1	0.000	0.000	0.000	0.000	0.000	0.000
		q=2	0.000	0.000	0.000	0.000	0.000	0.000
		q=3	0.000	0.000	0.000	0.000	0.000	0.000
		q=4	0.000	0.000	0.000	0.000	0.000	0.000
Hard palate	Male vs. Female	***q=0***	3.000	0.000	3.000	-1.083	1.159	0.000
		q=1	2.700	0.000	2.700	-2.022	2.081	0.000
		q=2	2.490	0.000	2.490	-2.159	2.208	0.000
		q=3	2.349	0.000	2.349	-2.133	2.178	0.000
		q=4	2.254	0.000	2.254	-2.086	2.128	0.000
Palatine Tonsils	Male vs. Female	***q=0***	0.000	0.000	0.000	0.000	0.000	0.000
		q=1	0.000	0.000	0.000	0.000	0.000	0.000
		q=2	0.000	0.000	0.000	0.000	0.000	0.000
		q=3	0.000	0.000	0.000	0.000	0.000	0.000
		q=4	0.000	0.000	0.000	0.000	0.000	0.000
Saliva	Male vs. Female	***q=0***	4.000	0.000	4.000	-2.003	2.315	0.000
		q=1	3.876	0.000	3.876	-2.336	2.508	0.000
		q=2	3.765	0.000	3.765	-2.507	2.638	0.000
		q=3	3.671	0.000	3.671	-2.525	2.640	0.000
		q=4	3.594	0.000	3.594	-2.502	2.609	0.000

Subgingival plaque	Male vs. Female	***q=0***	4.000	0.000	4.000	-0.289	0.293	0.000
		q=1	3.951	0.000	3.951	-0.888	0.982	0.000
		q=2	3.904	0.000	3.904	-1.142	1.248	0.000
		q=3	3.860	0.000	3.860	-1.254	1.357	0.000
		q=4	3.819	0.000	3.819	-1.308	1.403	0.000
Supragingival plaque	Male vs. Female	***q=0***	5.000	0.000	5.000	-1.173	1.243	0.000
		q=1	4.766	0.000	4.766	-1.957	2.040	0.000
		q=2	4.546	0.000	4.546	-2.203	2.281	0.000
		q=3	4.350	0.000	4.350	-2.246	2.320	0.000
		q=4	4.183	0.000	4.183	-2.237	2.308	0.000
Throat	Male vs. Female	***q=0***	1.000	0.000	1.000	-0.433	0.467	0.000
		q=1	1.000	0.000	1.000	-0.433	0.467	0.000
		q=2	1.000	0.000	1.000	-0.433	0.467	0.000
		q=3	1.000	0.000	1.000	-0.433	0.467	0.000
		q=4	1.000	0.000	1.000	-0.433	0.467	0.000
Tongue dorsum	Male vs. Female	***q=0***	6.000	0.000	6.000	-0.620	0.632	0.000
		q=1	5.653	0.000	5.653	-2.172	2.181	0.000
		q=2	5.388	0.000	5.388	-2.492	2.473	0.000
		q=3	5.195	0.000	5.195	-2.549	2.514	0.000
		q=4	5.057	0.000	5.057	-2.540	2.496	0.000
Left Antecubital fossa	Male vs. Female	***q=0***	8.000	0.000	8.000	-0.221	0.241	0.000
		q=1	7.696	0.000	7.696	-1.414	1.646	0.000
		q=2	7.441	0.000	7.441	-2.022	2.310	0.000
		q=3	7.230	0.000	7.230	-2.326	2.628	0.000
		q=4	7.054	0.000	7.054	-2.479	2.780	0.000
Right Antecubital fossa	Male vs. Female	***q=0***	21.000	0.000	21.000	-2.850	3.220	0.000
		q=1	19.296	0.000	19.296	-5.401	5.949	0.000
		q=2	17.837	0.000	17.837	-6.215	6.786	0.000
		q=3	16.668	0.000	16.668	-6.437	6.991	0.000
		q=4	15.764	0.000	15.764	-6.450	6.982	0.000
Left Retroauricular crease	Male vs. Female	***q=0***	6.000	0.000	6.000	-0.163	0.165	0.000
		q=1	5.869	0.000	5.869	-1.178	1.233	0.000
		q=2	5.738	0.000	5.738	-1.670	1.731	0.000
		q=3	5.610	0.000	5.610	-1.901	1.966	0.000
		q=4	5.489	0.000	5.489	-2.009	2.075	0.000
Right Retroauricular crease	Male vs. Female	***q=0***	4.000	0.000	4.000	-0.179	0.193	0.000
		q=1	3.987	0.000	3.987	-0.670	0.786	0.000
		q=2	3.974	0.000	3.974	-0.906	1.048	0.000
		q=3	3.962	0.000	3.962	-1.030	1.177	0.000
		q=4	3.950	0.000	3.950	-1.099	1.245	0.000
**Percentage of significant difference (%)**		***q=0***						100
		q=1						100
		q=2						100
		q=3						93.3
		q=4						93.3

Table S3B. The specificity diversity (SD) permutation (SDP) tests for the species category of unique species (US) in the female between both sexes

Microbiome	Treatments	Order	Male	Female	Delta	Lower (95%)	Upper (95%)	*p*-Value
Anterior nares	Male vs. Female	***q=0***	0.000	163.000	-163.000	-222.591	261.517	0.000
		q=1	0.000	136.643	-136.643	-223.200	254.344	0.002
		q=2	0.000	111.468	-111.468	-208.598	234.388	0.663
		q=3	0.000	92.428	-92.428	-189.007	210.422	0.831
		q=4	0.000	79.800	-79.800	-170.511	188.715	0.821
Stool	Male vs. Female	***q=0***	0.000	4.000	-4.000	-1.709	1.629	0.000
		q=1	0.000	3.684	-3.684	-1.675	1.659	0.000
		q=2	0.000	3.448	-3.448	-1.736	1.734	0.000
		q=3	0.000	3.288	-3.288	-1.770	1.776	0.000
		q=4	0.000	3.182	-3.182	-1.786	1.796	0.000
Attached Keratinized gingiva	Male vs. Female	***q=0***	0.000	18.000	-18.000	-24.465	26.671	0.000
		q=1	0.000	12.641	-12.641	-22.700	24.253	0.321
		q=2	0.000	8.699	-8.699	-20.435	21.565	0.791
		q=3	0.000	6.723	-6.723	-18.433	19.287	0.807
		q=4	0.000	5.771	-5.771	-16.919	17.609	0.796
Buccal mucosa	Male vs. Female	***q=0***	0.000	30.000	-30.000	-37.035	39.011	0.000
		q=1	0.000	21.943	-21.943	-34.672	36.035	0.012
		q=2	0.000	16.615	-16.615	-30.643	31.585	0.397
		q=3	0.000	13.835	-13.835	-26.901	27.585	0.508
		q=4	0.000	12.357	-12.357	-24.126	24.664	0.495
Hard palate	Male vs. Female	***q=0***	0.000	15.000	-15.000	-16.973	18.201	0.000
		q=1	0.000	12.919	-12.919	-14.548	15.433	0.000
		q=2	0.000	11.378	-11.378	-12.903	13.601	0.000
		q=3	0.000	10.315	-10.315	-11.699	12.279	0.003
		q=4	0.000	9.576	-9.576	-10.831	11.338	0.008
Palatine Tonsils	Male vs. Female	***q=0***	0.000	32.000	-32.000	-40.679	40.465	0.000
		q=1	0.000	24.426	-24.426	-36.928	36.628	0.000
		q=2	0.000	19.520	-19.520	-32.869	32.542	0.083
		q=3	0.000	16.894	-16.894	-29.133	28.820	0.192
		q=4	0.000	15.450	-15.450	-26.252	25.973	0.215
Saliva	Male vs. Female	***q=0***	0.000	53.000	-53.000	-54.163	60.049	0.000
		q=1	0.000	42.532	-42.532	-52.195	55.438	0.000
		q=2	0.000	34.616	-34.616	-48.067	50.080	0.004
		q=3	0.000	29.191	-29.191	-43.461	44.891	0.041
		q=4	0.000	25.532	-25.532	-39.649	40.781	0.123
Subgingival plaque	Male vs. Female	***q=0***	0.000	29.000	-29.000	-30.979	32.041	0.000
		q=1	0.000	21.058	-21.058	-28.335	28.856	0.000
		q=2	0.000	16.110	-16.110	-24.461	24.809	0.054
		q=3	0.000	13.365	-13.365	-21.271	21.562	0.157
		q=4	0.000	11.756	-11.756	-19.040	19.313	0.214
Supragingival plaque	Male vs. Female	***q=0***	0.000	21.000	-21.000	-22.473	23.185	0.000
		q=1	0.000	15.091	-15.091	-19.753	20.325	0.001
		q=2	0.000	11.907	-11.907	-16.916	17.400	0.033
		q=3	0.000	10.352	-10.352	-14.767	15.175	0.072
		q=4	0.000	9.498	-9.498	-13.310	13.663	0.088
Throat	Male vs. Female	***q=0***	0.000	12.000	-12.000	-13.236	13.476	0.000
		q=1	0.000	9.066	-9.066	-13.247	13.186	0.000
		q=2	0.000	7.309	-7.309	-11.953	11.799	0.107

		q=3	0.000	6.372	-6.372	-10.743	10.569	0.228
		q=4	0.000	5.839	-5.839	-9.859	9.691	0.245
Tongue dorsum	Male vs. Female	***q=0***	0.000	18.000	-18.000	-18.719	19.249	0.000
		q=1	0.000	13.576	-13.576	-19.063	19.245	0.002
		q=2	0.000	10.730	-10.730	-17.201	17.233	0.134
		q=3	0.000	9.073	-9.073	-15.373	15.333	0.246
		q=4	0.000	8.085	-8.085	-14.024	13.949	0.287
Left Antecubital fossa	Male vs. Female	***q=0***	0.000	20.000	-20.000	-15.082	17.644	0.000
		q=1	0.000	16.156	-16.156	-13.859	15.815	0.000
		q=2	0.000	14.322	-14.322	-12.227	13.611	0.000
		q=3	0.000	13.376	-13.376	-10.869	11.902	0.000
		q=4	0.000	12.786	-12.786	-9.899	10.743	0.000
Right Antecubital fossa	Male vs. Female	***q=0***	0.000	34.000	-34.000	-53.111	58.363	0.000
		q=1	0.000	30.636	-30.636	-50.373	54.984	0.000
		q=2	0.000	27.272	-27.272	-46.886	50.896	0.006
		q=3	0.000	24.498	-24.498	-43.133	46.604	0.153
		q=4	0.000	22.489	-22.489	-39.680	42.725	0.288
Left Retroauricular crease	Male vs. Female	***q=0***	0.000	25.000	-25.000	-30.771	34.397	0.000
		q=1	0.000	21.219	-21.219	-28.226	31.095	0.000
		q=2	0.000	18.000	-18.000	-25.852	28.302	0.014
		q=3	0.000	15.573	-15.573	-23.574	25.768	0.084
		q=4	0.000	13.846	-13.846	-21.753	23.780	0.171
Right Retroauricular crease	Male vs. Female	***q=0***	0.000	25.000	-25.000	-29.258	30.822	0.000
		q=1	0.000	17.410	-17.410	-27.484	28.361	0.028
		q=2	0.000	12.844	-12.844	-24.264	24.784	0.456
		q=3	0.000	10.728	-10.728	-21.420	21.754	0.518
		q=4	0.000	9.700	-9.700	-19.348	19.594	0.489
**Percentage of significant difference (%)**		***q=0***						100
		q=1						93.3
		q=2						46.7
		q=3						26.7
		q=4						20

Table 3C. The specificity diversity (SD) permutation (SDP) tests for the species category of enriched species (ES) in the male between both sexes

Microbiome	Treatments	Order	Male	Female	Delta	Lower (95%)	Upper (95%)	*p*-Value
Anterior nares	Male vs. Female	***q=0***	9.000	9.000	0.000	0.000	0.000	0.000
		q=1	8.530	5.594	2.936	-1.449	1.593	0.000
		q=2	8.086	4.858	3.228	-2.014	2.190	0.001
		q=3	7.699	4.514	3.185	-2.250	2.432	0.005
		q=4	7.379	4.305	3.074	-2.360	2.542	0.011
Stool	Male vs. Female	***q=0***	4.000	4.000	0.000	-0.061	0.063	0.001
		q=1	3.493	1.186	2.307	-1.876	1.876	0.003
		q=2	3.050	1.064	1.986	-2.048	2.047	0.041
		q=3	2.740	1.048	1.692	-1.928	1.926	0.081
		q=4	2.545	1.043	1.503	-1.804	1.803	0.100
Attached Keratinized gingiva	Male vs. Female	***q=0***	3.000	3.000	0.000	0.000	0.000	0.000
		q=1	2.893	2.408	0.485	-0.931	0.957	0.365
		q=2	2.804	2.172	0.631	-1.144	1.167	0.352
		q=3	2.730	2.059	0.671	-1.184	1.202	0.338
		q=4	2.671	1.991	0.681	-1.184	1.197	0.327
Buccal mucosa	Male vs. Female	***q=0***	2.000	2.000	0.000	0.000	0.000	0.000
		q=1	1.999	1.955	0.044	-0.114	0.122	0.303
		q=2	1.998	1.913	0.085	-0.184	0.198	0.293
		q=3	1.996	1.876	0.120	-0.228	0.244	0.262
		q=4	1.995	1.844	0.151	-0.256	0.274	0.238
Hard palate	Male vs. Female	***q=0***	4.000	4.000	0.000	0.000	0.000	0.000
		q=1	3.836	2.786	1.051	-0.588	0.584	0.000
		q=2	3.691	2.409	1.282	-0.853	0.849	0.002
		q=3	3.569	2.265	1.303	-0.959	0.956	0.004
		q=4	3.468	2.189	1.279	-1.002	0.999	0.009
Palatine Tonsils	Male vs. Female	***q=0***	1.000	1.000	0.000	0.000	0.000	0.000
		q=1	1.000	1.000	0.000	0.000	0.000	0.000
		q=2	1.000	1.000	0.000	0.000	0.000	0.000
		q=3	1.000	1.000	0.000	0.000	0.000	0.000
		q=4	1.000	1.000	0.000	0.000	0.000	0.000
Saliva	Male vs. Female	***q=0***	1.000	1.000	0.000	0.000	0.000	0.000
		q=1	1.000	1.000	0.000	0.000	0.000	0.000
		q=2	1.000	1.000	0.000	0.000	0.000	0.000
		q=3	1.000	1.000	0.000	0.000	0.000	0.000
		q=4	1.000	1.000	0.000	0.000	0.000	0.000
Subgingival plaque	Male vs. Female	***q=0***	3.000	3.000	0.000	0.000	0.000	0.000
		q=1	2.756	1.695	1.061	-1.103	1.160	0.036
		q=2	2.589	1.515	1.074	-1.260	1.302	0.093
		q=3	2.478	1.428	1.050	-1.298	1.327	0.121
		q=4	2.402	1.383	1.019	-1.306	1.328	0.134
Supragingival plaque	Male vs. Female	***q=0***	2.000	2.000	0.000	0.000	0.000	0.000
		q=1	1.832	1.157	0.676	-0.765	0.755	0.073
		q=2	1.710	1.068	0.641	-0.877	0.866	0.169
		q=3	1.628	1.052	0.576	-0.862	0.851	0.217
		q=4	1.575	1.046	0.529	-0.830	0.819	0.235
Throat	Male vs. Female	***q=0***	1.000	1.000	0.000	0.000	0.000	0.000
		q=1	1.000	1.000	0.000	0.000	0.000	0.000
		q=2	1.000	1.000	0.000	0.000	0.000	0.000

		q=3	1.000	1.000	0.000	0.000	0.000	0.000
		q=4	1.000	1.000	0.000	0.000	0.000	0.000
Tongue dorsum	Male vs. Female	***q=0***	15.000	15.000	0.000	0.000	0.000	0.000
		q=1	13.759	5.034	8.725	-3.765	3.579	0.000
		q=2	12.569	2.997	9.572	-5.242	4.992	0.000
		q=3	11.532	2.450	9.082	-5.503	5.259	0.000
		q=4	10.694	2.238	8.457	-5.330	5.109	0.000
Left Antecubital fossa	Male vs. Female	***q=0***	35.000	35.000	0.000	0.000	0.000	0.000
		q=1	33.908	20.854	13.054	-2.806	3.303	0.000
		q=2	32.882	15.224	17.658	-4.422	5.148	0.000
		q=3	31.936	12.154	19.782	-5.422	6.259	0.000
		q=4	31.078	10.392	20.685	-6.060	6.948	0.000
Right Antecubital fossa	Male vs. Female	***q=0***	31.000	31.000	0.000	0.000	0.000	0.000
		q=1	28.946	15.472	13.474	-4.231	4.358	0.000
		q=2	27.179	10.280	16.899	-6.201	6.336	0.000
		q=3	25.725	8.117	17.607	-7.094	7.224	0.000
		q=4	24.557	7.130	17.426	-7.391	7.524	0.000
Left Retroauricular crease	Male vs. Female	***q=0***	17.000	17.000	0.000	0.000	0.000	0.000
		q=1	16.008	9.314	6.694	-2.599	2.625	0.000
		q=2	15.174	6.925	8.249	-3.890	3.901	0.000
		q=3	14.492	6.051	8.441	-4.494	4.486	0.000
		q=4	13.942	5.658	8.284	-4.722	4.701	0.000
Right Retroauricular crease	Male vs. Female	***q=0***	32.000	32.000	0.000	0.000	0.000	0.000
		q=1	29.472	19.984	9.488	-2.380	2.551	0.000
		q=2	27.752	17.193	10.559	-2.894	3.071	0.000
		q=3	26.563	15.655	10.907	-3.179	3.345	0.000
		q=4	25.703	14.679	11.024	-3.388	3.542	0.000
**Percentage of significant difference (%)**		***q=0***						100
		q=1						80
		q=2						73.3
		q=3						66.7
		q=4						66.7

Table 3D. The specificity diversity (SD) permutation (SDP) tests for the species category of enriched species (ES) in the female between both sexes

Microbiome	Treatments	Order	Male	Female	Delta	Lower (95%)	Upper (95%)	*p*-Value
Anterior nares	Male vs. Female	***q=0***	1.000	1.000	0.000	-0.122	0.126	0.004
		q=1	1.000	1.000	0.000	-0.122	0.126	0.004
		q=2	1.000	1.000	0.000	-0.122	0.126	0.004
		q=3	1.000	1.000	0.000	-0.122	0.126	0.004
		q=4	1.000	1.000	0.000	-0.122	0.126	0.004
Stool	Male vs. Female	***q=0***	3.000	3.000	0.000	-0.106	0.108	0.003
		q=1	2.129	2.945	-0.816	-0.964	0.968	0.103
		q=2	1.736	2.888	-1.152	-1.260	1.261	0.076
		q=3	1.580	2.833	-1.253	-1.371	1.368	0.069
		q=4	1.510	2.781	-1.271	-1.414	1.409	0.066
Attached Keratinized gingiva	Male vs. Female	***q=0***	1.000	1.000	0.000	0.000	0.000	0.000
		q=1	1.000	1.000	0.000	0.000	0.000	0.000
		q=2	1.000	1.000	0.000	0.000	0.000	0.000
		q=3	1.000	1.000	0.000	0.000	0.000	0.000
		q=4	1.000	1.000	0.000	0.000	0.000	0.000
Buccal mucosa	Male vs. Female	***q=0***	10.000	10.000	0.000	-0.063	0.061	0.001
		q=1	3.690	9.098	-5.408	-2.590	2.606	0.000
		q=2	2.426	8.239	-5.813	-3.369	3.348	0.000
		q=3	2.064	7.502	-5.438	-3.510	3.460	0.000
		q=4	1.918	6.923	-5.005	-3.441	3.374	0.001
Hard palate	Male vs. Female	***q=0***	3.000	3.000	0.000	0.000	0.000	0.000
		q=1	2.052	2.943	-0.891	-0.846	0.919	0.041
		q=2	1.951	2.886	-0.935	-1.140	1.227	0.125
		q=3	1.904	2.830	-0.926	-1.259	1.349	0.207
		q=4	1.869	2.777	-0.908	-1.311	1.401	0.241
Palatine Tonsils	Male vs. Female	***q=0***	2.000	2.000	0.000	0.000	0.000	0.000
		q=1	1.592	1.902	-0.309	-0.488	0.513	0.238
		q=2	1.409	1.820	-0.411	-0.683	0.718	0.287
		q=3	1.330	1.756	-0.425	-0.747	0.784	0.327
		q=4	1.294	1.708	-0.415	-0.762	0.800	0.345
Saliva	Male vs. Female	***q=0***	7.000	7.000	0.000	-0.171	0.179	0.008
		q=1	5.033	6.502	-1.469	-1.143	1.227	0.005
		q=2	4.219	6.235	-2.016	-1.451	1.540	0.000
		q=3	3.729	6.070	-2.341	-1.643	1.730	0.000
		q=4	3.422	5.952	-2.530	-1.772	1.855	0.000
Subgingival plaque	Male vs. Female	***q=0***	3.000	3.000	0.000	0.000	0.000	0.000
		q=1	1.926	2.856	-0.929	-0.685	0.691	0.006
		q=2	1.549	2.719	-1.170	-0.991	0.997	0.009
		q=3	1.424	2.601	-1.177	-1.070	1.075	0.020
		q=4	1.372	2.505	-1.133	-1.065	1.069	0.026
Supragingival plaque	Male vs. Female	***q=0***	1.000	1.000	0.000	0.000	0.000	0.000
		q=1	1.000	1.000	0.000	0.000	0.000	0.000
		q=2	1.000	1.000	0.000	0.000	0.000	0.000
		q=3	1.000	1.000	0.000	0.000	0.000	0.000
		q=4	1.000	1.000	0.000	0.000	0.000	0.000
Throat	Male vs. Female	***q=0***	1.000	1.000	0.000	0.000	0.000	0.000
		q=1	1.000	1.000	0.000	0.000	0.000	0.000
		q=2	1.000	1.000	0.000	0.000	0.000	0.000

		q=3	1.000	1.000	0.000	0.000	0.000	0.000
		q=4	1.000	1.000	0.000	0.000	0.000	0.000
Tongue dorsum	Male vs. Female	***q=0***	12.000	12.000	0.000	0.000	0.000	0.000
		q=1	9.345	11.314	-1.969	-1.204	1.165	0.000
		q=2	8.092	10.732	-2.640	-1.803	1.745	0.002
		q=3	7.397	10.253	-2.855	-2.154	2.088	0.004
		q=4	6.953	9.863	-2.910	-2.379	2.308	0.009
Left Antecubital fossa	Male vs. Female	***q=0***	2.000	2.000	0.000	0.000	0.000	0.000
		q=1	1.362	1.931	-0.568	-0.707	0.704	0.113
		q=2	1.203	1.869	-0.667	-0.933	0.920	0.176
		q=3	1.157	1.818	-0.662	-1.001	0.982	0.244
		q=4	1.139	1.778	-0.639	-1.017	0.995	0.293
Right Antecubital fossa	Male vs. Female	***q=0***	0.000	0.000	0.000	0.000	0.000	0.000
		q=1	0.000	0.000	0.000	0.000	0.000	0.000
		q=2	0.000	0.000	0.000	0.000	0.000	0.000
		q=3	0.000	0.000	0.000	0.000	0.000	0.000
		q=4	0.000	0.000	0.000	0.000	0.000	0.000
Left Retroauricular crease	Male vs. Female	***q=0***	12.000	12.000	0.000	-0.061	0.063	0.001
		q=1	7.247	10.571	-3.324	-1.209	1.254	0.000
		q=2	6.033	9.824	-3.791	-1.291	1.318	0.000
		q=3	5.281	9.406	-4.125	-1.390	1.406	0.000
		q=4	4.793	9.144	-4.351	-1.503	1.514	0.000
Right Retroauricular crease	Male vs. Female	***q=0***	7.000	7.000	0.000	-0.138	0.140	0.005
		q=1	2.910	6.177	-3.267	-2.150	2.166	0.001
		q=2	2.665	5.595	-2.930	-2.132	2.123	0.001
		q=3	2.566	5.222	-2.656	-1.953	1.933	0.002
		q=4	2.509	4.983	-2.475	-1.824	1.799	0.004
**Percentage of significant difference (%)**		***q=0***						100
		q=1						80
		q=2						73.3
		q=3						73.3
		q=4						73.3

Table 3E. The specificity diversity (SD) permutation (SDP) tests for the species category of "all species with significant differences in specificity" between both sexes

Microbiome	Treatments	Order	Male	Female	Delta	Lower (95%)	Upper (95%)	*p*-Value
Anterior nares	Male vs. Female	*q=0*	12.000	173.000	-161.000	-222.551	261.571	0.000
		q=1	9.826	140.285	-130.459	-168.713	182.836	0.000
		q=2	9.039	112.438	-103.398	-88.121	90.836	0.000
		q=3	8.483	92.858	-84.375	-53.219	54.550	0.000
		q=4	8.065	80.483	-72.418	-39.905	41.091	0.000
Stool	Male vs. Female	*q=0*	16.000	11.000	5.000	-4.527	4.403	0.019
		q=1	9.667	7.155	2.512	-8.169	7.934	0.565
		q=2	7.236	6.408	0.828	-7.693	7.477	0.836
		q=3	5.869	5.931	-0.063	-6.717	6.536	0.982
		q=4	5.121	5.594	-0.473	-5.953	5.798	0.867
Attached Keratinized gingiva	Male vs. Female	*q=0*	7.000	22.000	-15.000	-24.417	26.681	0.019
		q=1	5.042	12.815	-7.772	-17.488	18.245	0.539
		q=2	4.368	8.428	-4.060	-12.652	12.881	0.533
		q=3	3.999	6.750	-2.751	-10.271	10.362	0.540
		q=4	3.780	6.013	-2.233	-8.989	9.035	0.559
Buccal mucosa	Male vs. Female	*q=0*	12.000	42.000	-30.000	-37.038	39.012	0.000
		q=1	4.517	22.270	-17.753	-20.694	21.027	0.004
		q=2	3.622	15.504	-11.882	-12.148	12.183	0.019
		q=3	3.368	12.697	-9.330	-9.358	9.334	0.036
		q=4	3.247	11.136	-7.889	-8.166	8.115	0.050
Hard palate	Male vs. Female	*q=0*	10.000	22.000	-12.000	-16.928	18.232	0.006
		q=1	5.552	14.192	-8.641	-9.327	9.860	0.011
		q=2	4.786	10.438	-5.651	-5.230	5.532	0.018
		q=3	4.431	8.744	-4.314	-3.893	4.116	0.026
		q=4	4.217	7.856	-3.639	-3.342	3.532	0.035
Palatine Tonsils	Male vs. Female	*q=0*	3.000	35.000	-32.000	-40.679	40.465	0.000
		q=1	2.309	16.722	-14.412	-24.616	24.378	0.215
		q=2	2.042	8.805	-6.763	-12.420	12.289	0.322
		q=3	1.914	6.384	-4.470	-8.166	8.073	0.297
		q=4	1.839	5.439	-3.601	-6.549	6.470	0.299
Saliva	Male vs. Female	*q=0*	12.000	61.000	-49.000	-54.100	60.306	0.000
		q=1	6.649	31.543	-24.893	-37.188	38.107	0.058
		q=2	4.188	19.379	-15.191	-20.490	20.606	0.064
		q=3	3.280	15.425	-12.145	-14.163	14.238	0.023
		q=4	2.912	13.776	-10.864	-11.566	11.648	0.015
Subgingival plaque	Male vs. Female	*q=0*	10.000	35.000	-25.000	-30.970	32.036	0.000
		q=1	7.505	17.073	-9.569	-17.766	18.081	0.380
		q=2	6.289	10.273	-3.984	-9.878	10.083	0.522
		q=3	5.536	7.902	-2.366	-7.075	7.258	0.558
		q=4	5.063	6.839	-1.775	-5.917	6.089	0.587
Supragingival plaque	Male vs. Female	*q=0*	8.000	24.000	-16.000	-22.491	23.273	0.000
		q=1	5.460	12.862	-7.403	-14.407	14.501	0.410
		q=2	4.132	7.698	-3.565	-8.875	8.803	0.478
		q=3	3.469	5.700	-2.231	-6.487	6.410	0.513
		q=4	3.130	4.849	-1.719	-5.383	5.313	0.541
Throat	Male vs. Female	*q=0*	3.000	14.000	-11.000	-13.235	13.509	0.000
		q=1	1.924	8.597	-6.673	-10.855	10.808	0.212
		q=2	1.600	5.997	-4.397	-7.784	7.728	0.324

		q=3	1.480	4.873	-3.393	-6.162	6.133	0.328
		q=4	1.425	4.319	-2.893	-5.312	5.298	0.323
Tongue dorsum	Male vs. Female	***q=0***	33.000	45.000	-12.000	-18.709	19.251	0.034
		q=1	25.770	18.879	6.891	-9.760	9.196	0.164
		q=2	21.574	14.698	6.876	-8.331	7.717	0.092
		q=3	18.749	13.242	5.507	-7.594	7.034	0.147
		q=4	16.784	12.421	4.364	-7.019	6.517	0.222
Left Antecubital fossa	Male vs. Female	***q=0***	45.000	57.000	-12.000	-15.065	17.647	0.004
		q=1	41.399	26.435	14.964	-9.146	10.229	0.000
		q=2	39.502	16.529	22.973	-8.170	9.155	0.000
		q=3	37.985	12.301	25.683	-8.413	9.456	0.000
		q=4	36.700	10.235	26.465	-8.759	9.844	0.000
Right Antecubital fossa	Male vs. Female	***q=0***	52.000	65.000	-13.000	-52.768	58.390	1.000
		q=1	42.476	41.317	1.160	-24.202	25.482	0.986
		q=2	36.916	26.344	10.572	-14.604	15.076	0.165
		q=3	33.523	18.670	14.852	-12.289	12.605	0.016
		q=4	31.273	15.223	16.050	-11.359	11.635	0.004
Left Retroauricular crease	Male vs. Female	***q=0***	35.000	54.000	-19.000	-30.772	34.402	0.060
		q=1	26.884	21.062	5.821	-10.699	11.331	0.370
		q=2	23.774	14.847	8.927	-7.349	7.450	0.012
		q=3	21.779	13.087	8.692	-6.653	6.613	0.006
		q=4	20.415	12.292	8.123	-6.257	6.159	0.007
Right Retroauricular crease	Male vs. Female	***q=0***	43.000	64.000	-21.000	-29.241	30.821	0.000
		q=1	33.728	36.151	-2.423	-8.432	8.762	0.749
		q=2	30.713	27.694	3.019	-4.866	5.112	0.237
		q=3	28.932	23.593	5.339	-4.245	4.464	0.015
		q=4	27.767	21.158	6.609	-4.145	4.346	0.001
**Percentage of significant difference (%)**		***q=0***						86.7
		q=1						26.7
		q=2						33.3
		q=3						53.3
		q=4						46.7

Table 3F. The specificity diversity (SD) permutation (SDP) tests for the species category of "all species (with or without specificity differences)" between both sexes

Microbiome	Treatments	Order	Male	Female	Delta	Lower (95%)	Upper (95%)	*p*-Value
Anterior nares	Male vs. Female	***q=0***	13126.000	11773.000	1353.000	-475.391	2811.793	0.420
		q=1	6329.332	6181.112	148.220	-929.418	1605.211	0.859
		q=2	3751.436	3524.418	227.018	-772.111	1008.208	0.643
		q=3	2789.311	2443.387	345.924	-596.422	735.525	0.318
		q=4	2329.476	1941.958	387.518	-494.814	599.637	0.171
Stool	Male vs. Female	***q=0***	6882.000	6972.000	-90.000	-402.494	512.586	0.704
		q=1	3806.671	3797.089	9.581	-454.670	506.618	0.972
		q=2	2657.297	2566.551	90.746	-394.962	428.407	0.672
		q=3	2122.722	1993.389	129.334	-341.016	367.780	0.504
		q=4	1824.958	1673.728	151.230	-303.391	327.039	0.370
Attached Keratinized gingiva	Male vs. Female	***q=0***	9620.000	8698.000	922.000	-739.963	1157.005	0.073
		q=1	4859.955	4279.804	580.150	-783.882	859.176	0.170
		q=2	2971.216	2553.991	417.225	-576.611	589.274	0.173
		q=3	2186.814	1867.073	319.741	-439.123	441.756	0.160
		q=4	1786.682	1526.520	260.163	-363.420	363.029	0.162
Buccal mucosa	Male vs. Female	***q=0***	11559.000	11800.000	-241.000	-617.037	1167.635	0.646
		q=1	5764.671	5891.310	-126.639	-656.241	787.609	0.741
		q=2	3569.488	3604.797	-35.309	-551.547	597.227	0.906
		q=3	2652.468	2680.614	-28.145	-468.307	494.189	0.901
		q=4	2180.210	2218.112	-37.901	-419.731	437.920	0.858
Hard palate	Male vs. Female	***q=0***	12543.000	11654.000	889.000	-868.617	1413.509	0.171
		q=1	6211.338	5828.897	382.441	-727.617	879.752	0.349
		q=2	3810.913	3593.332	217.581	-562.853	628.577	0.480
		q=3	2848.542	2696.276	152.266	-448.540	492.902	0.531
		q=4	2377.080	2252.586	124.494	-381.224	416.637	0.545
Palatine Tonsils	Male vs. Female	***q=0***	12757.000	12588.000	169.000	-901.903	1058.677	0.755
		q=1	6587.183	6355.509	231.674	-859.930	894.758	0.626
		q=2	4129.406	3889.255	240.151	-679.201	686.759	0.499
		q=3	3073.996	2882.093	191.903	-538.211	539.937	0.490
		q=4	2542.535	2382.905	159.630	-449.954	450.519	0.501
Saliva	Male vs. Female	***q=0***	12404.000	12206.000	198.000	-312.975	1467.623	0.854
		q=1	6429.712	6261.833	167.879	-533.840	810.695	0.651
		q=2	4045.747	3935.519	110.229	-503.559	593.411	0.702
		q=3	3044.238	2987.285	56.953	-430.986	481.769	0.800
		q=4	2534.809	2511.052	23.756	-377.519	415.076	0.906
Subgingival plaque	Male vs. Female	***q=0***	11215.000	11187.000	28.000	-679.179	1106.807	0.958
		q=1	5787.958	6104.975	-317.017	-698.372	805.082	0.430
		q=2	3751.012	4100.177	-349.166	-573.515	620.870	0.251
		q=3	2857.373	3199.287	-341.914	-494.871	527.963	0.194
		q=4	2379.665	2707.616	-327.950	-441.813	468.529	0.162
Supragingival plaque	Male vs. Female	***q=0***	10559.000	10434.000	125.000	-500.627	646.133	0.691
		q=1	5348.673	5618.479	-269.807	-622.562	640.973	0.404
		q=2	3465.391	3764.727	-299.336	-602.622	594.179	0.338
		q=3	2669.147	2934.831	-265.684	-540.042	527.783	0.346
		q=4	2254.122	2483.021	-228.899	-483.207	471.403	0.364
Throat	Male vs. Female	***q=0***	12930.000	12547.000	383.000	-1198.015	1992.233	0.683
		q=1	6693.580	6168.936	524.644	-1001.181	1178.219	0.365
		q=2	4137.908	3700.627	437.281	-696.698	742.647	0.252

		q=3	3070.811	2736.363	334.448	-520.918	544.190	0.235
		q=4	2536.555	2271.363	265.193	-425.338	442.762	0.244
Tongue dorsum	Male vs. Female	***q=0***	10372.000	9987.000	385.000	-755.718	1101.316	0.492
		q=1	5365.979	5006.608	359.370	-580.834	661.472	0.271
		q=2	3511.698	3222.215	289.483	-470.835	499.435	0.240
		q=3	2715.395	2481.524	233.871	-399.328	415.550	0.266
		q=4	2297.728	2099.388	198.340	-352.138	363.796	0.280
Left Antecubital fossa	Male vs. Female	***q=0***	14471.000	10714.000	3757.000	-1038.211	4069.351	0.031
		q=1	7576.912	6418.477	1158.435	-1354.959	2509.338	0.335
		q=2	4130.633	3722.717	407.916	-1285.809	1652.825	0.597
		q=3	2766.426	2421.572	344.853	-1000.930	1164.548	0.533
		q=4	2166.665	1819.382	347.284	-796.705	902.600	0.433
Right Antecubital fossa	Male vs. Female	***q=0***	14487.000	13107.000	1380.000	-1331.544	2227.946	0.182
		q=1	6999.896	6326.860	673.036	-1246.627	1514.226	0.353
		q=2	3996.475	3527.824	468.650	-903.669	986.930	0.334
		q=3	2850.222	2456.775	393.447	-661.740	701.022	0.280
		q=4	2320.731	1951.672	369.059	-532.603	557.295	0.189
Left Retroauricular crease	Male vs. Female	***q=0***	13968.000	11072.000	2896.000	-1624.151	3058.627	0.035
		q=1	7335.761	6537.996	797.765	-1449.682	1974.922	0.389
		q=2	4043.999	3804.710	239.289	-1191.079	1352.185	0.729
		q=3	2743.790	2519.524	224.267	-886.280	961.457	0.643
		q=4	2169.037	1923.480	245.557	-698.395	750.683	0.513
Right Retroauricular crease	Male vs. Female	***q=0***	13691.000	11912.000	1779.000	-1055.681	2006.903	0.041
		q=1	6466.961	5804.147	662.814	-1017.567	1279.862	0.292
		q=2	3673.556	3202.522	471.034	-738.337	817.521	0.251
		q=3	2632.241	2194.870	437.371	-549.302	590.397	0.136
		q=4	2156.338	1715.120	441.219	-449.382	479.245	0.055
**Percentage of significant difference (%)**		***q=0***						20%
		q=1						0
		q=2						0
		q=3						0
		q=4						0

Table S4. Numbers of species in different categories classified by the SP (specificity permutation) tests in the HMP datasets (*P*-value=0.05, with FDR control)

Sites	With significant difference				Total of Significantly Different Species	Total of Insignificantly Different Species	Total Species
	US in Male	US in Female	ES in Male	ES in Female			
Anterior nares	2	163	9	1	175	16400	16575
Stool	9	4	4	3	20	8028	8048
Attached Keratinized gingiva	3	18	3	1	25	11491	11516
Buccal mucosa	0	30	2	10	42	14277	14319
Hard palate	3	15	4	3	25	14800	14825
Palatine Tonsils	0	32	1	2	35	15070	15105
Saliva	4	53	1	7	65	15029	15094
Subgingival plaque	4	29	3	3	39	13247	13286
Supragingival plaque	5	21	2	1	29	12299	12328
Throat	1	12	1	1	15	15866	15881
Tongue dorsum	6	18	15	12	51	11934	11985
Left Antecubital fossa	8	20	35	2	65	17644	17709
Left Retroauricular crease	21	34	31	0	86	17322	17408
Right Antecubital fossa	6	25	17	12	60	17421	17481
Right Retroauricular crease	4	25	32	7	68	16376	16444
**Total**	76	499	128	65	800		

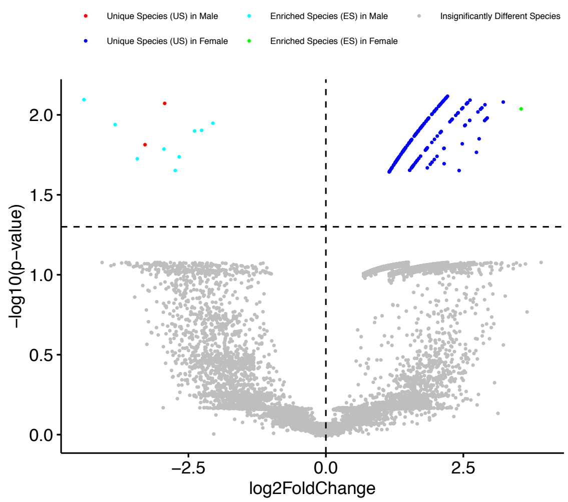

Anterior_nares

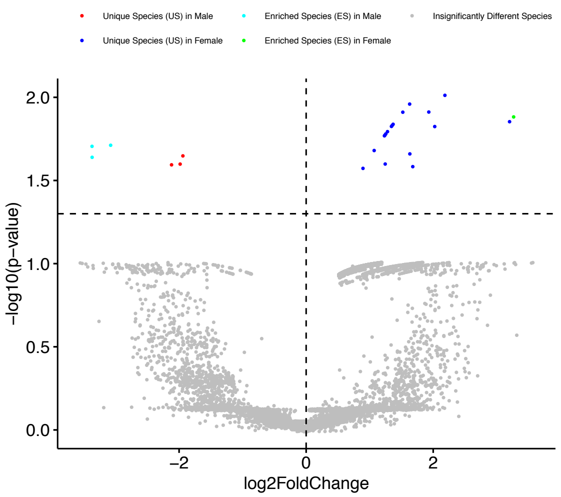

Attached_Keratinized_gingiva

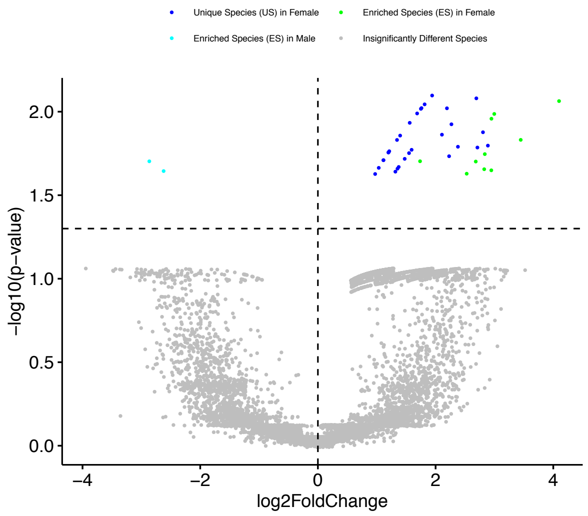

Buccal_mucosa

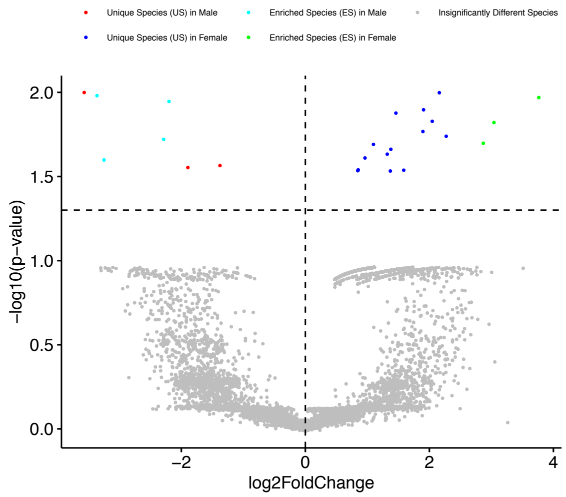

Hard_palate

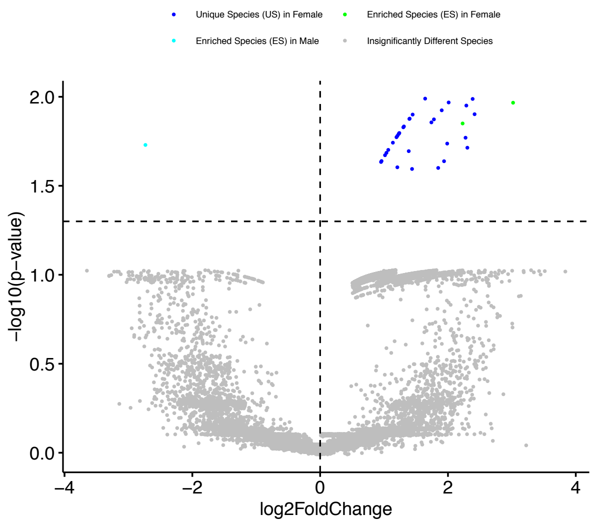

Palatine_Tonsils

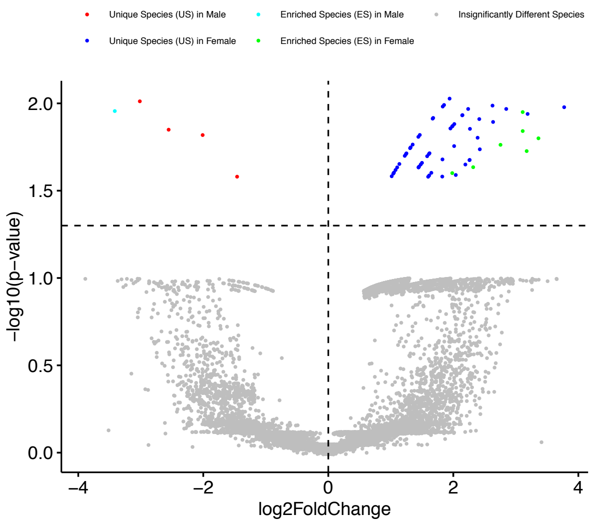

Saliva

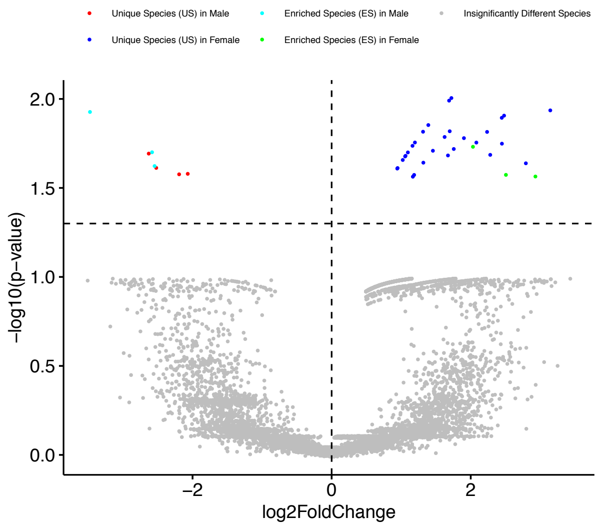

Subgingival_plaque

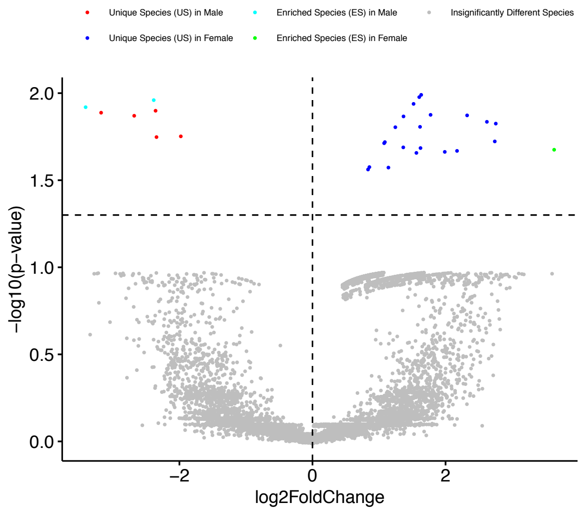

Supragingival_plaque

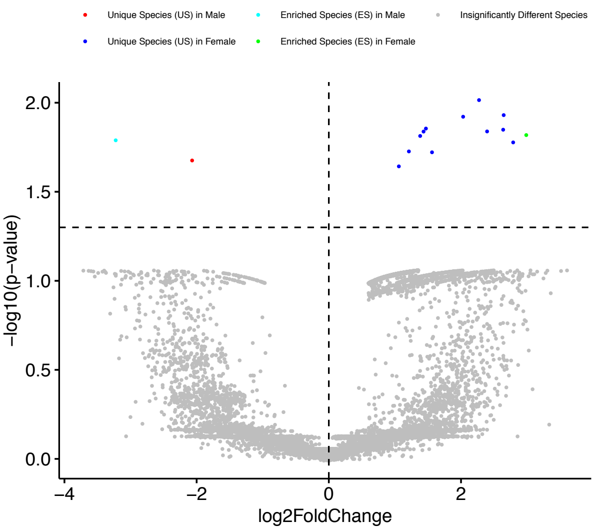

Throat

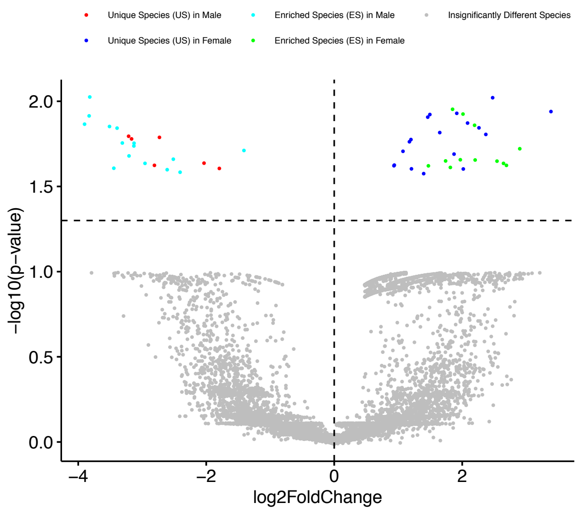

Tongue_dorsum

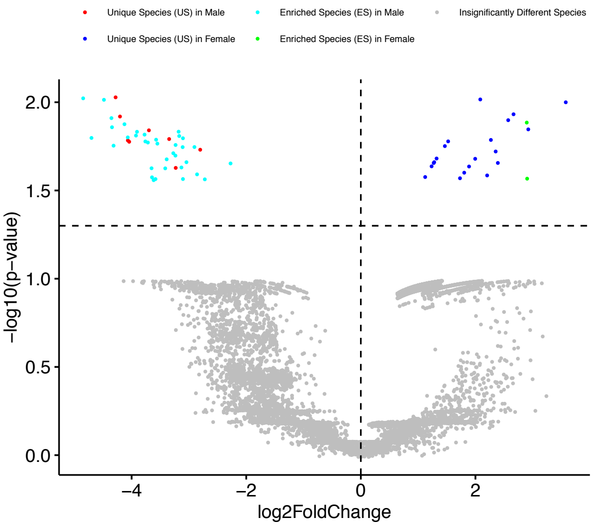

Left_Antecubital_fossa

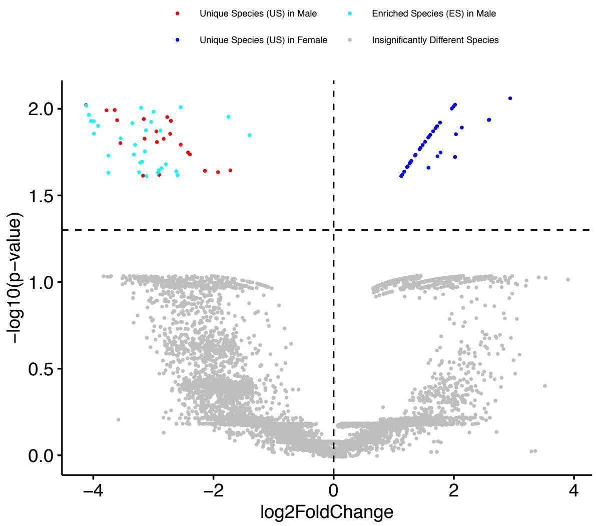

Right_Antecubital_fossa

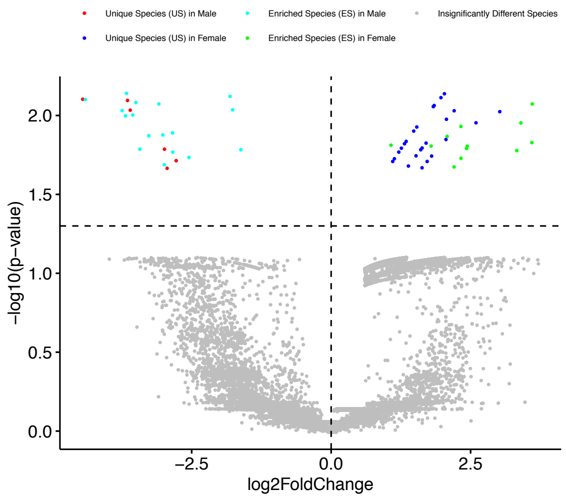

Left_Retroauricular_crease

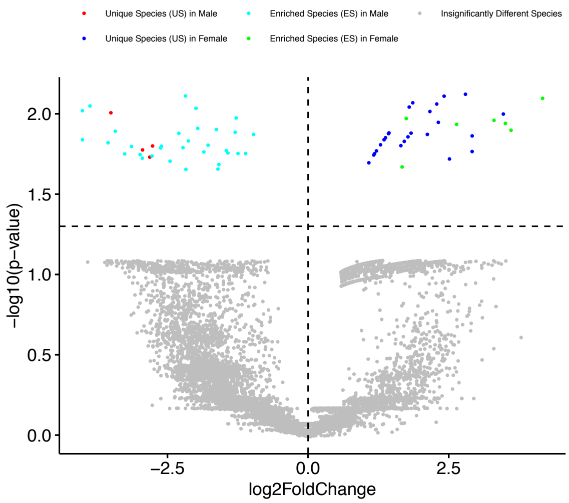

Right_Retroauricular_crease

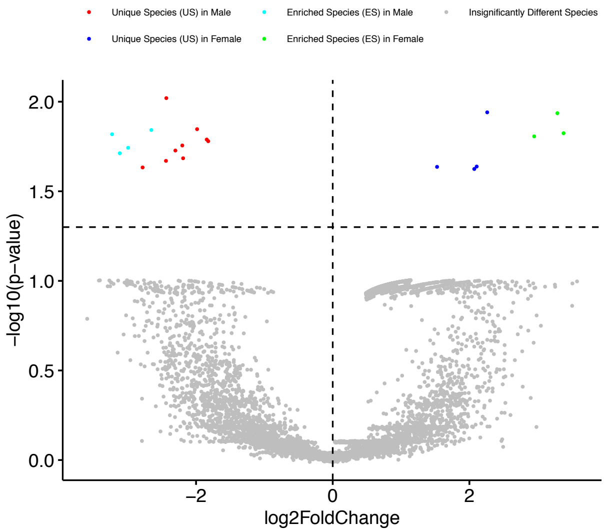

Stool

Fig S1A. The volcano graphs of the species groups: US (unique species) in the male (red dots) or female (blue dots), ES (enriched species) in the male (cyan dots) or female (green dots), and insignificantly different species (grey points) based on the specificity permutation (SP) tests. All 15 sites are included here, and 4 of which were also duplicated in the main article. The X-axis represents the log-transformation of the specificity fold change between male and female, where fold change=S(male)/S(female) (S represents specificity of species); Y-axis represents the negative log-transformation of the *P*-value from SP tests of the specificity differences between male and female cohorts. The vertical dotted line at X=0 represents fold change=1 [*i.e.* S(male)=S(female)], the points in the right side of this dotted line represent species with S(male)/S(female)>1 [*i.e.* S(male)>S(female)], the left points represents species with S(male)/S(female)<1 [*i.e.* S(male)<S(female)]. The horizontal dotted line represents *P*-value=0.05 [–log10*(0.05)=1.301], the points above the line represent species specificity with significant differences between male and female, and the points below represent species of non-significant differences in specificity. Therefore, the grey points represent species of non-significant differences in species specificity between male and female, cyan points represent significant enriched species in male, green points represent significant enriched species in female, red and blue points represent unique species in male and female, respectively. See **Fig S1B** below for some of the directly drawn volano graphs using specificity values, without using log(fold-changes)].

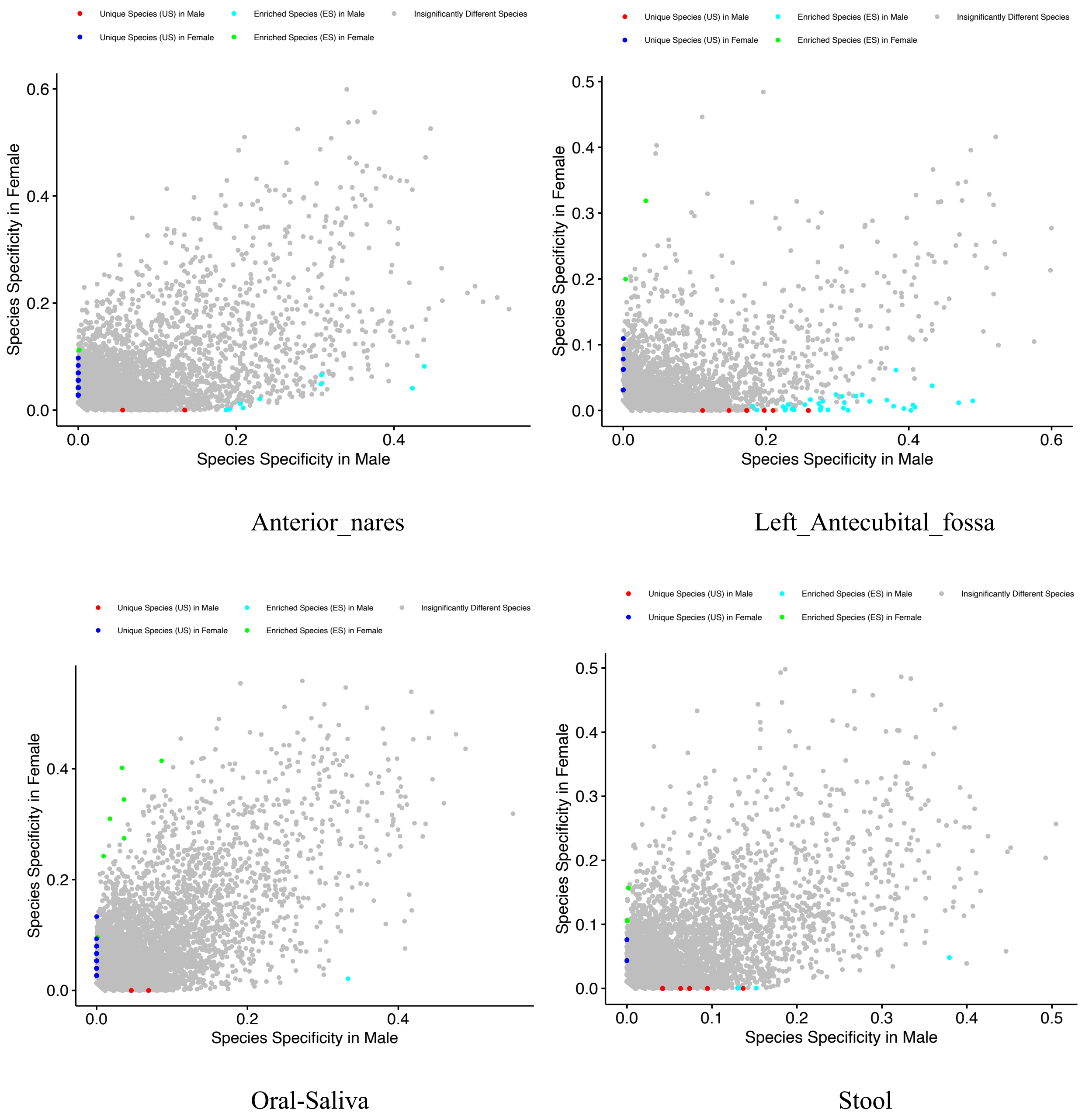

Fig S1B. The volcano graphs (drawn directly using species specificity without using log(fold-changes) of the specificity ratios) of the species groups: US (unique species) in the male (red dots) or female (blue dots), ES (enriched species) in the male (cyan dots) or female (green dots), and insignificantly different species (grey points) based on the specificity permutation (SP) tests. Only the graphs for four sites are illustrated here for demonstrative purpose.

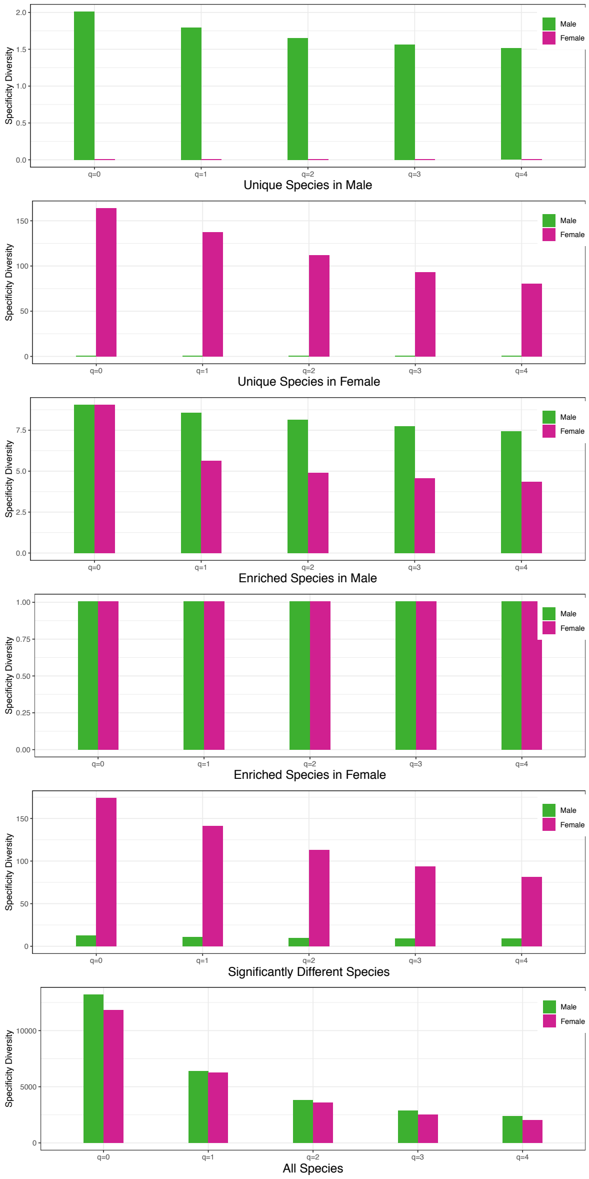

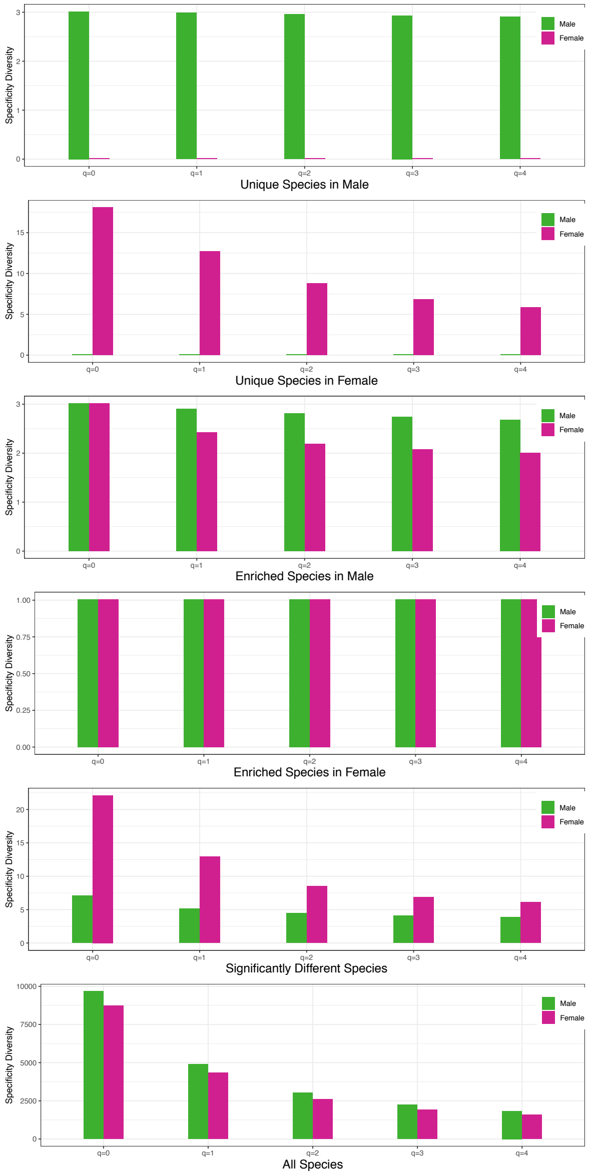

Anterior_nares

Attached_Keratinized_gingiva

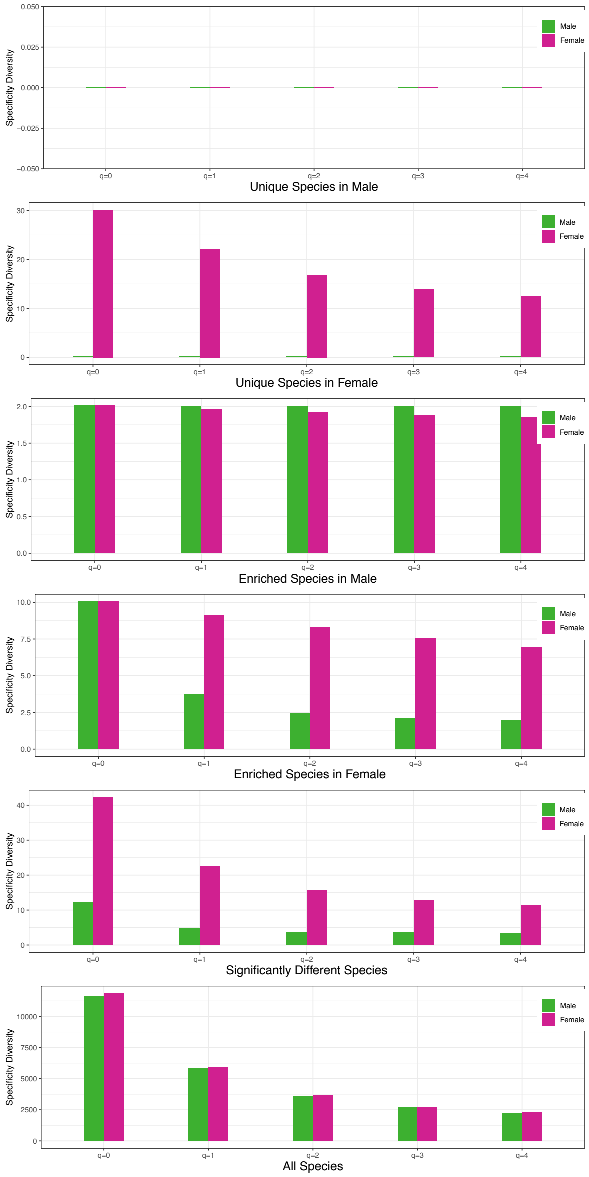

Buccal_mucosa

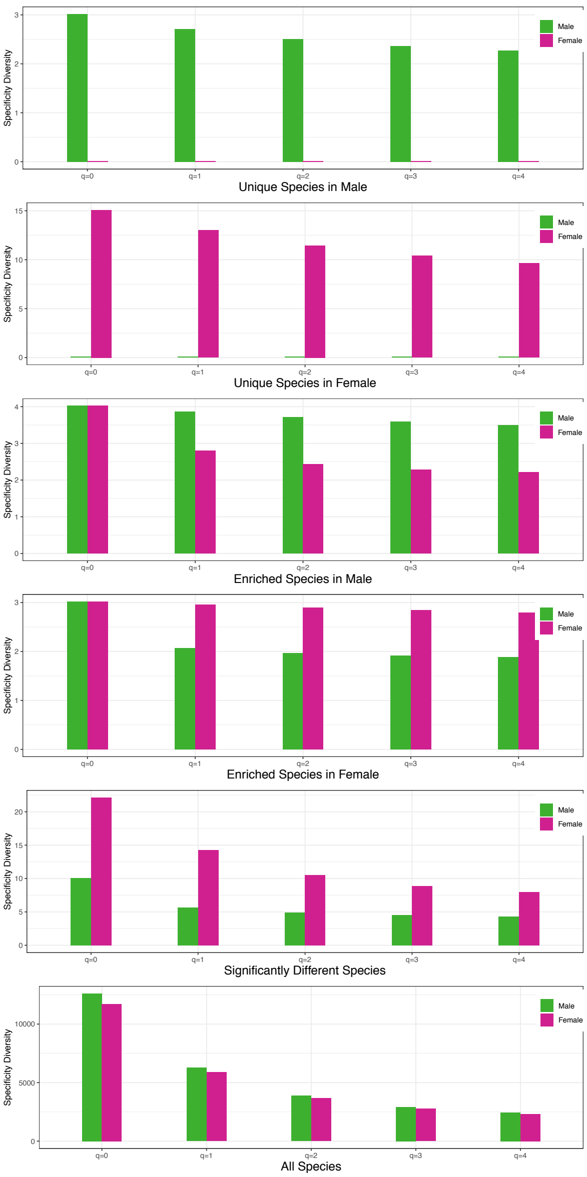

Hard_palate

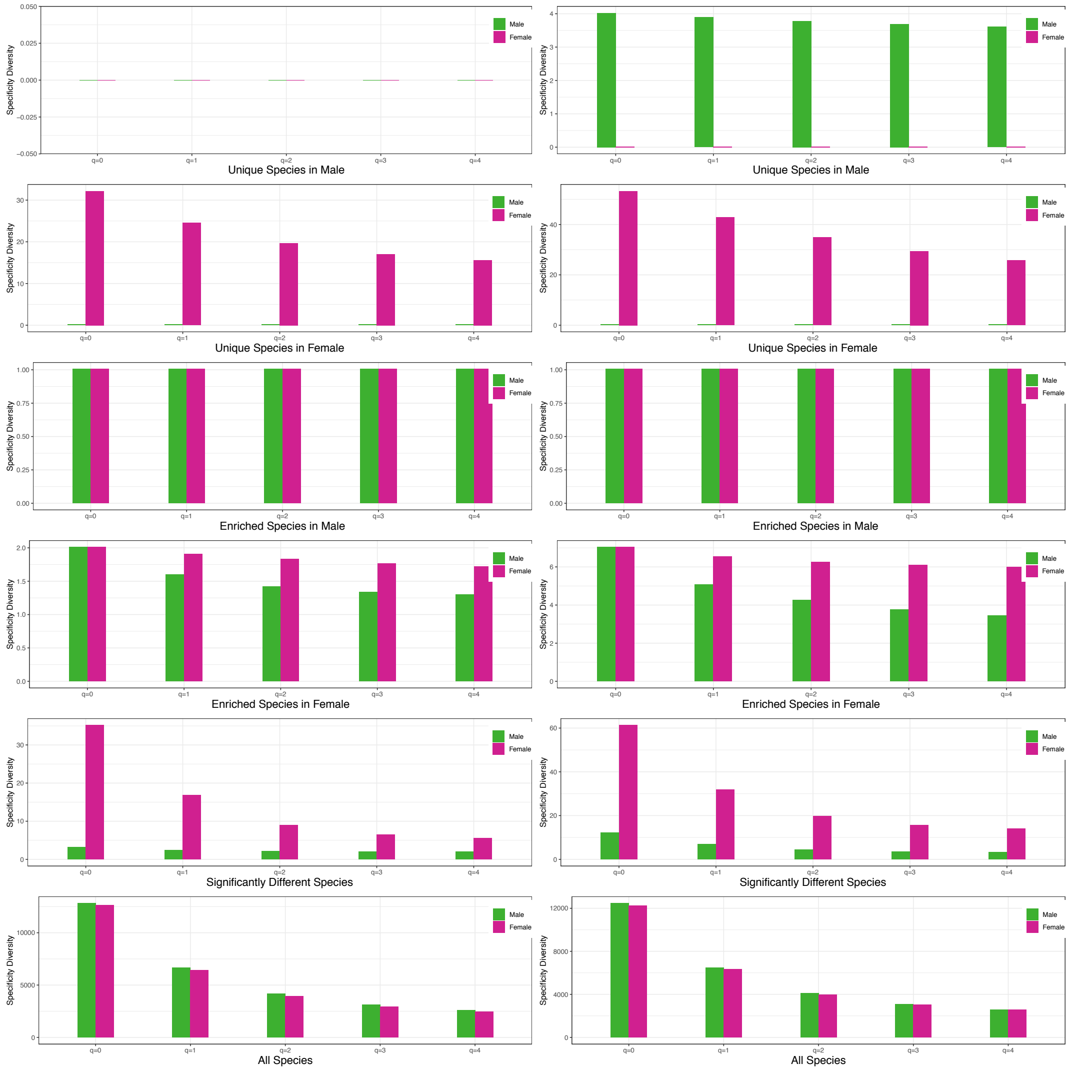

Palatine_Tonsils

Saliva

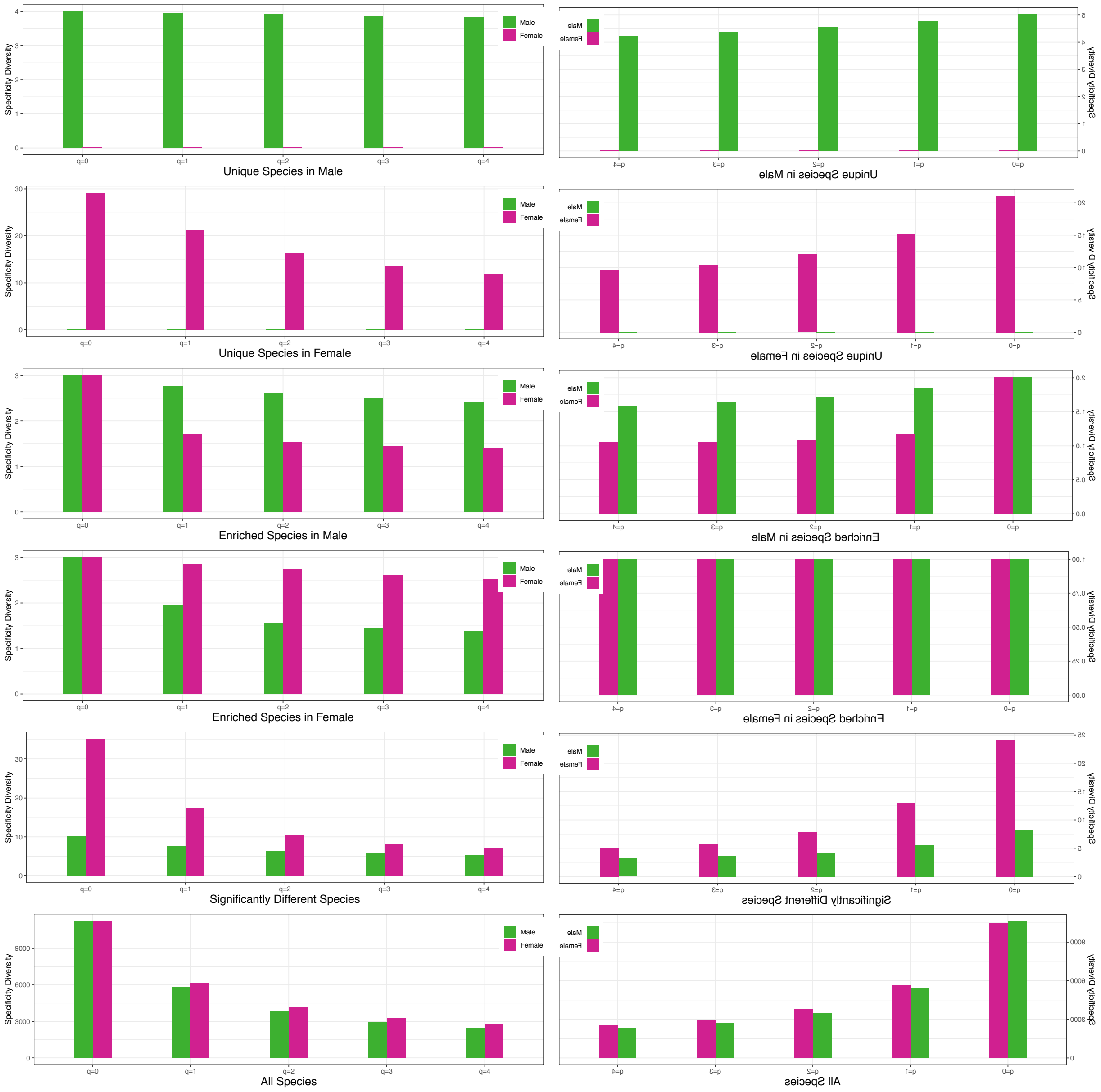

Subgingival_plaque Supragingival_plaque

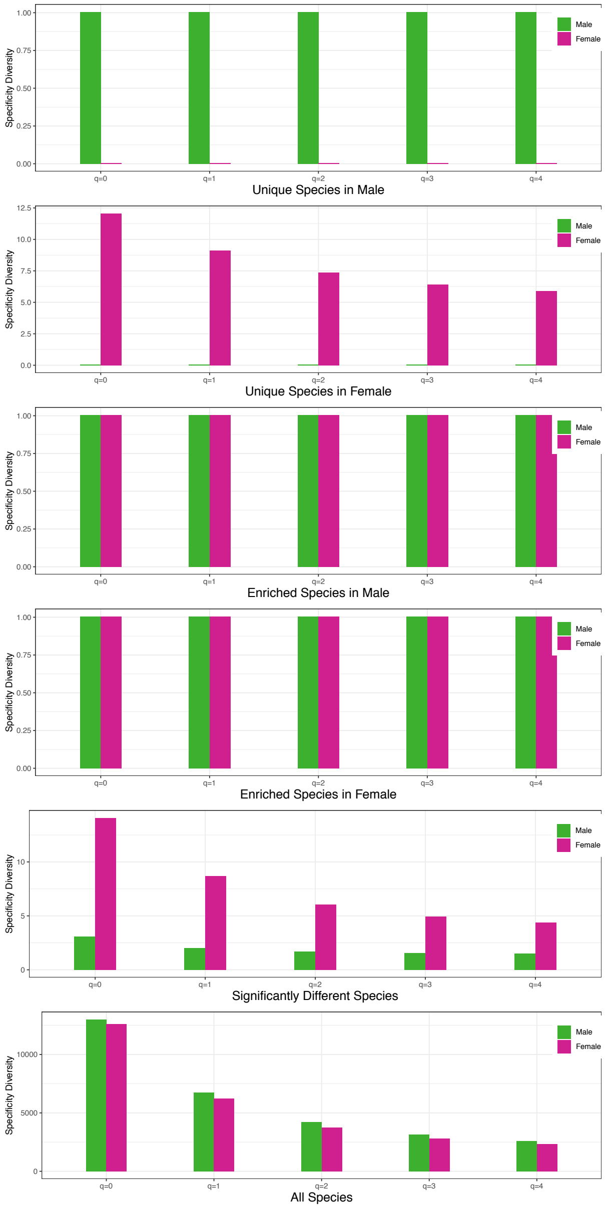

Throat

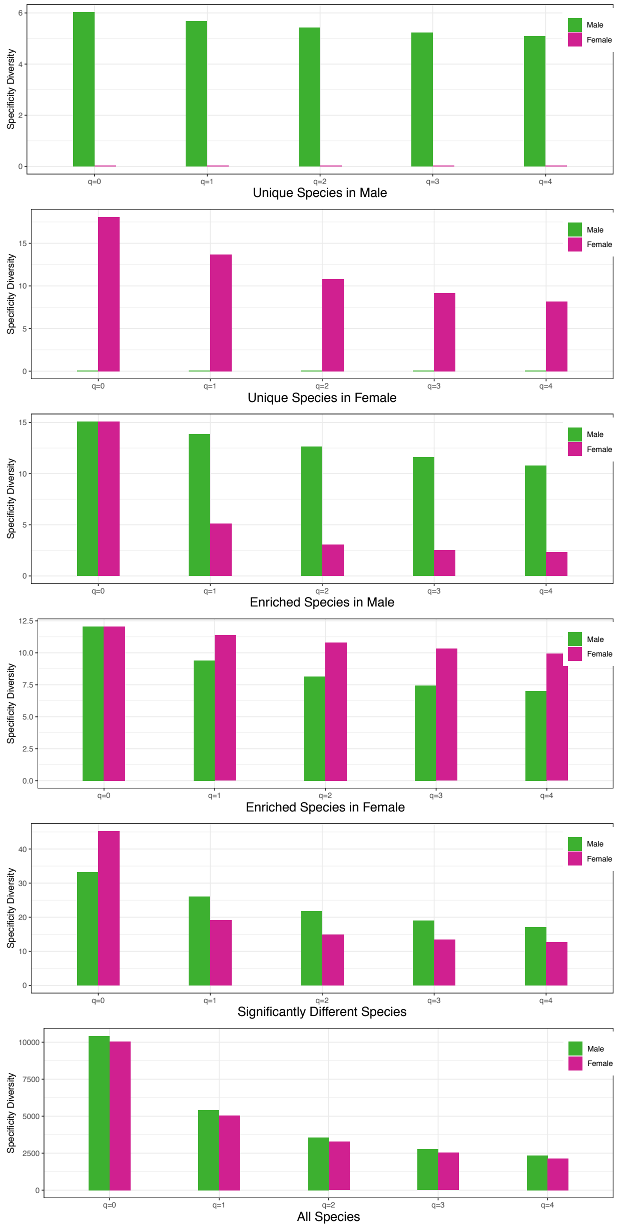

Tongue_dorsum

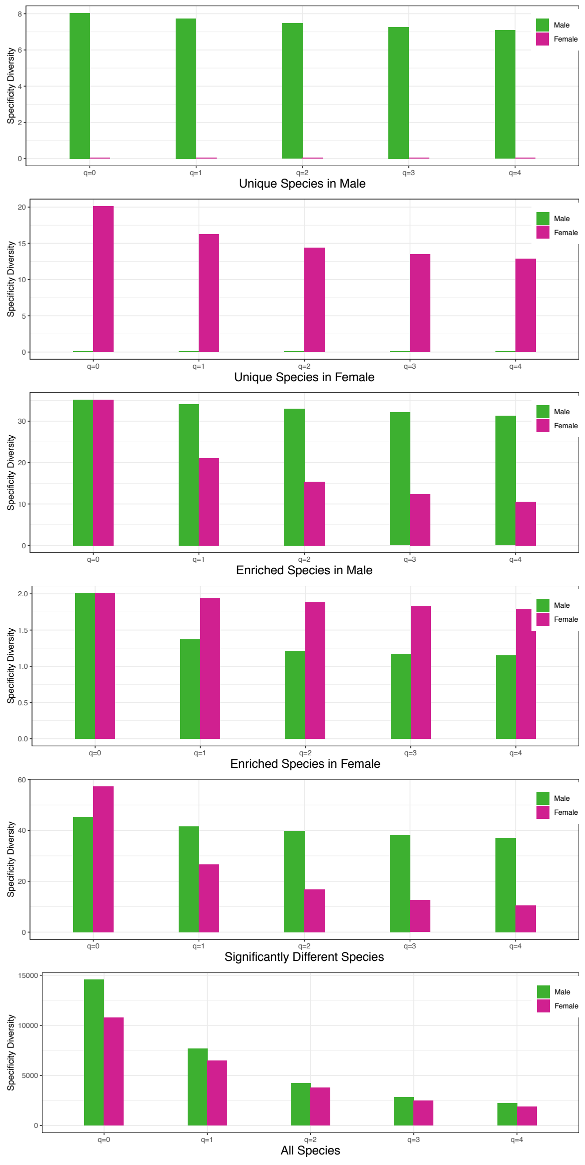

Left_Antecubital_fossa

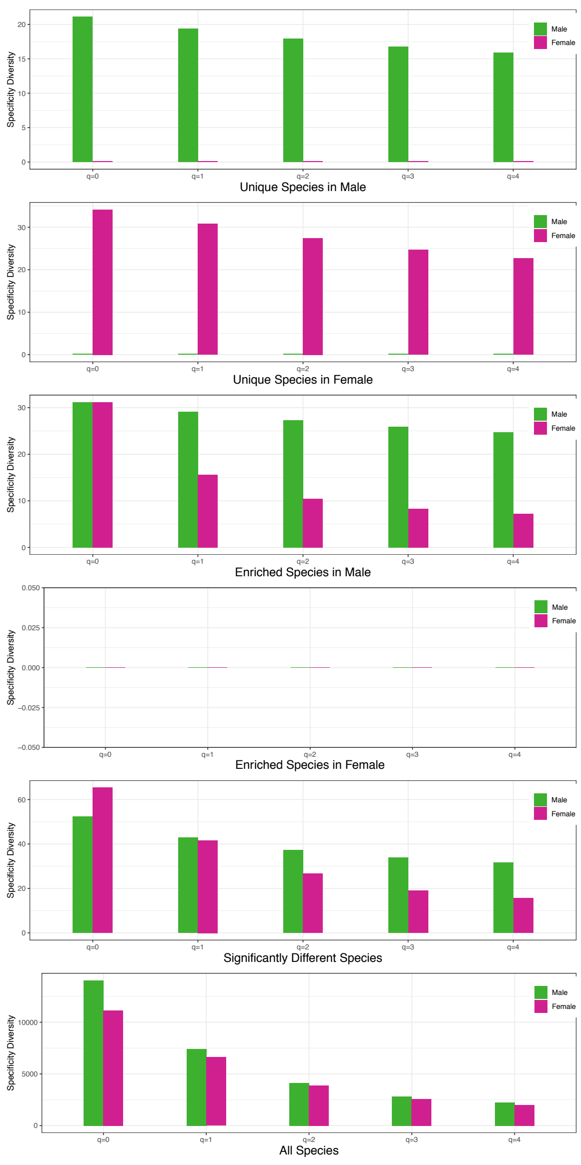

Right_Antecubital_fossa

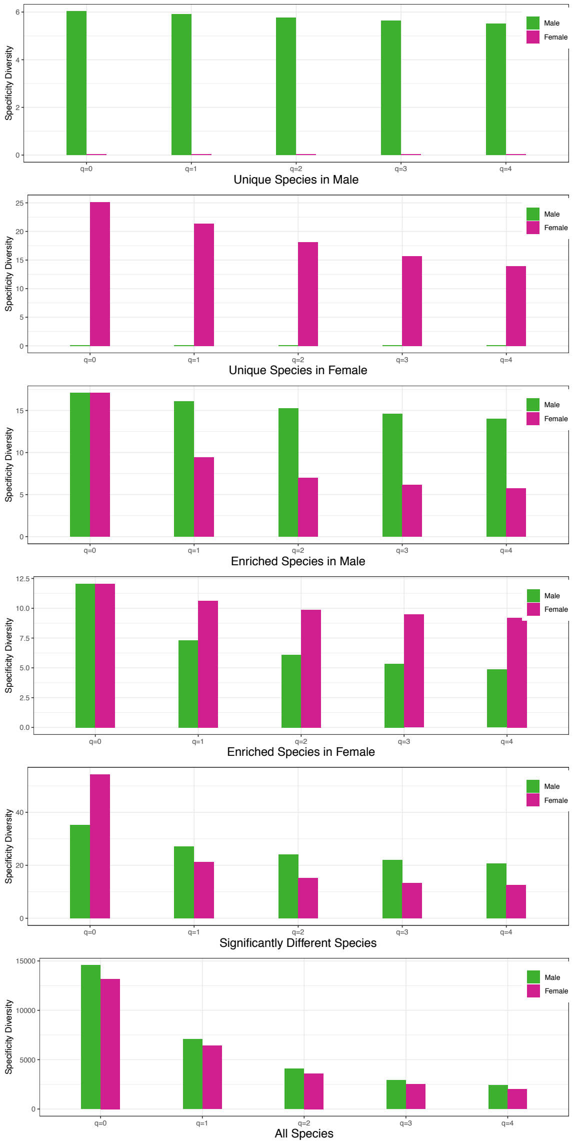

Left_Retroauricular_crease

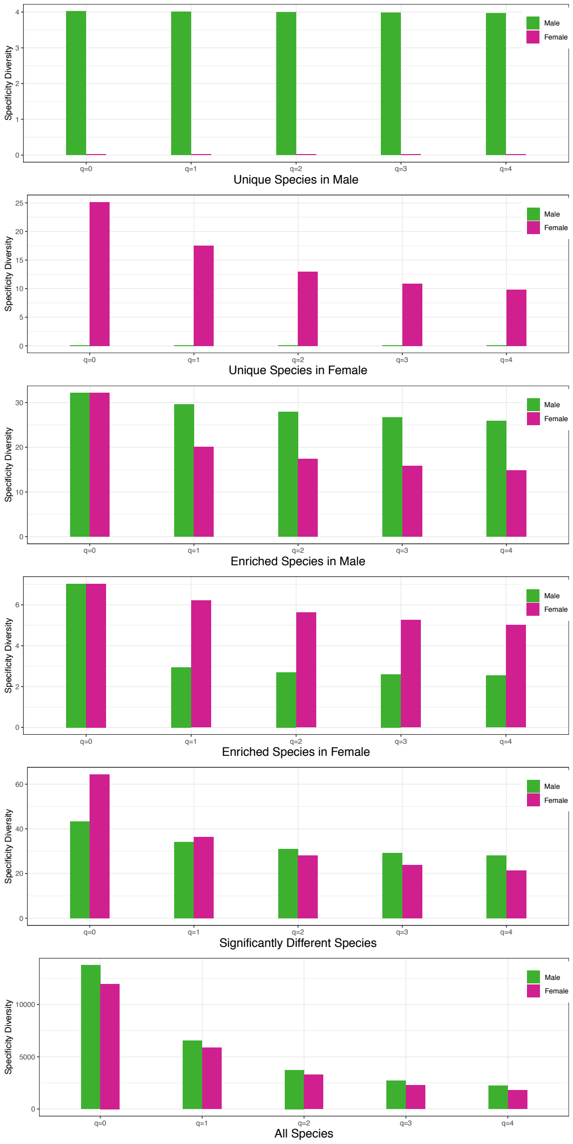

Right_Retroauricular_crease

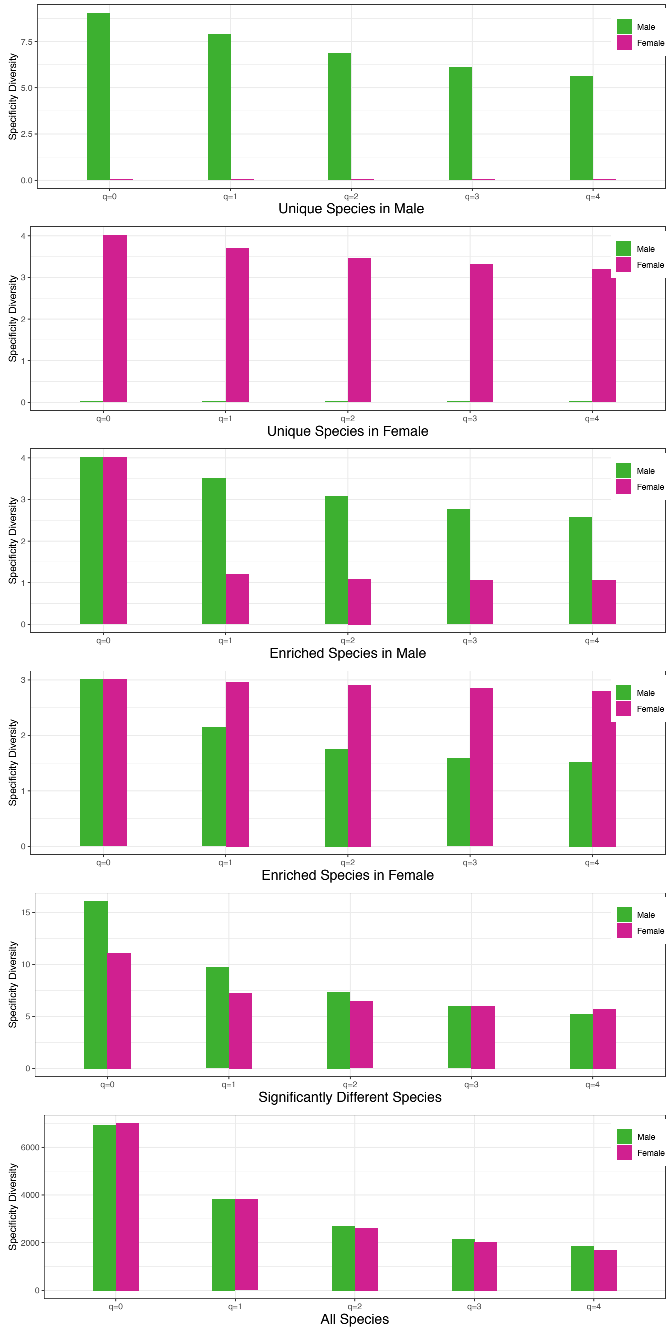

Stool

Fig S2. The specificity diversity (SD) for the 15 body sites of the HMP (human microbiome project), two of which (anterior nares and stool) were also displayed in the main mansucript: Y-axis shows the SD at different diversity order (*q*=0-4, X-axis) for each of the six species categories (from top to bottom plot) including unique species (US) in male, US in female, enriched species (ES) in male, ES in female, species with significant differences, and all species (with + without differences).

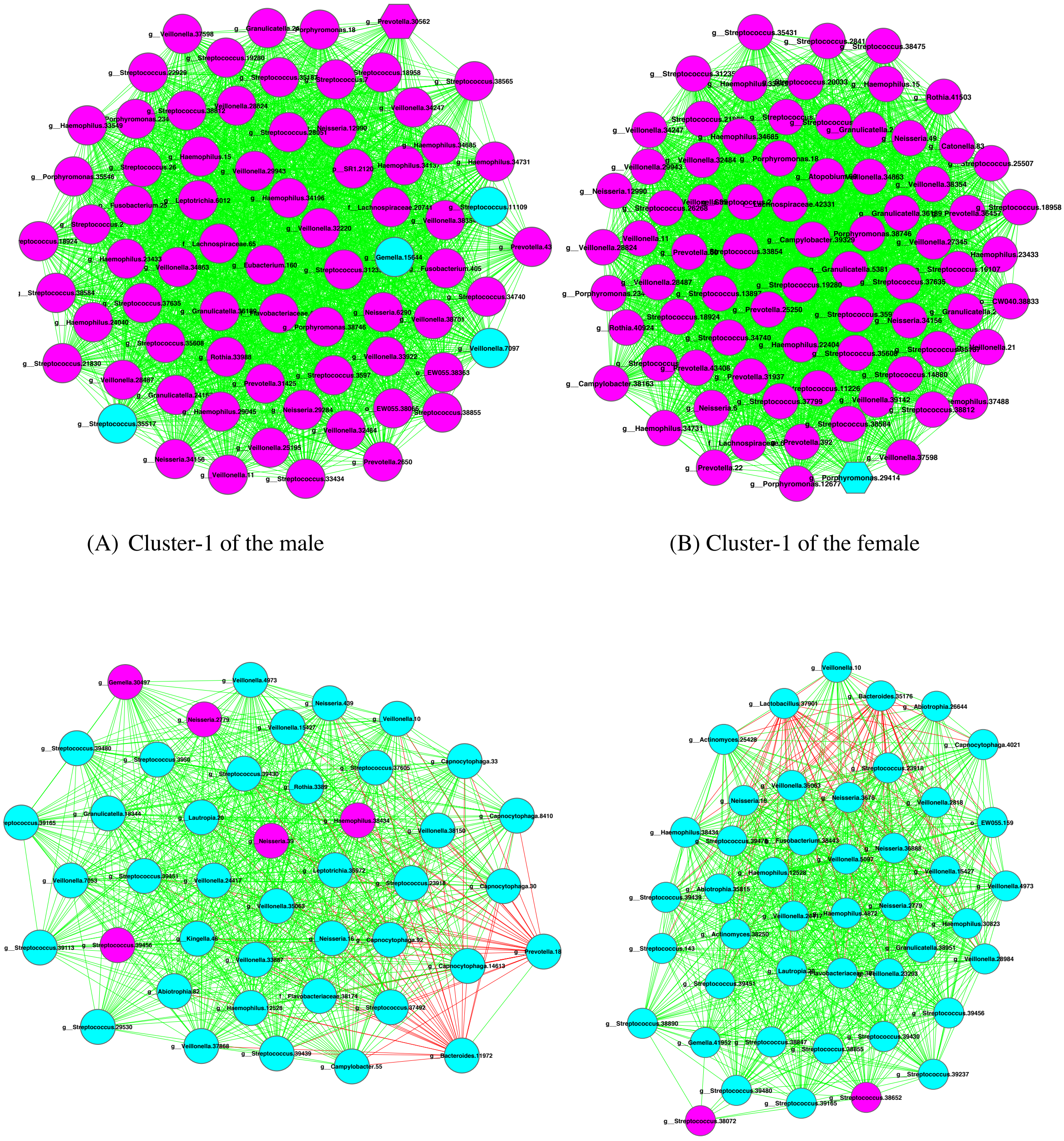

(A) Cluster-1 of the male

(B) Cluster-1 of the female

(C) Cluster-2 of the male

(D) Cluster-2 of the female

Fig S3. The top two strongest connected clusters in the SSNs (species specificity co-dependency network) of the male and female based on the HMP datasets: two findings are obvious: (*i*) both the male and female exhibited the same patterns as explained below; (*ii*) the strongest cluster consists of core nodes (pink color) except for a few nodes (periphery in cyan), the 2nd stongest cluster consists of periphery nodes (cyan color) except for a few core nodes (pink color).

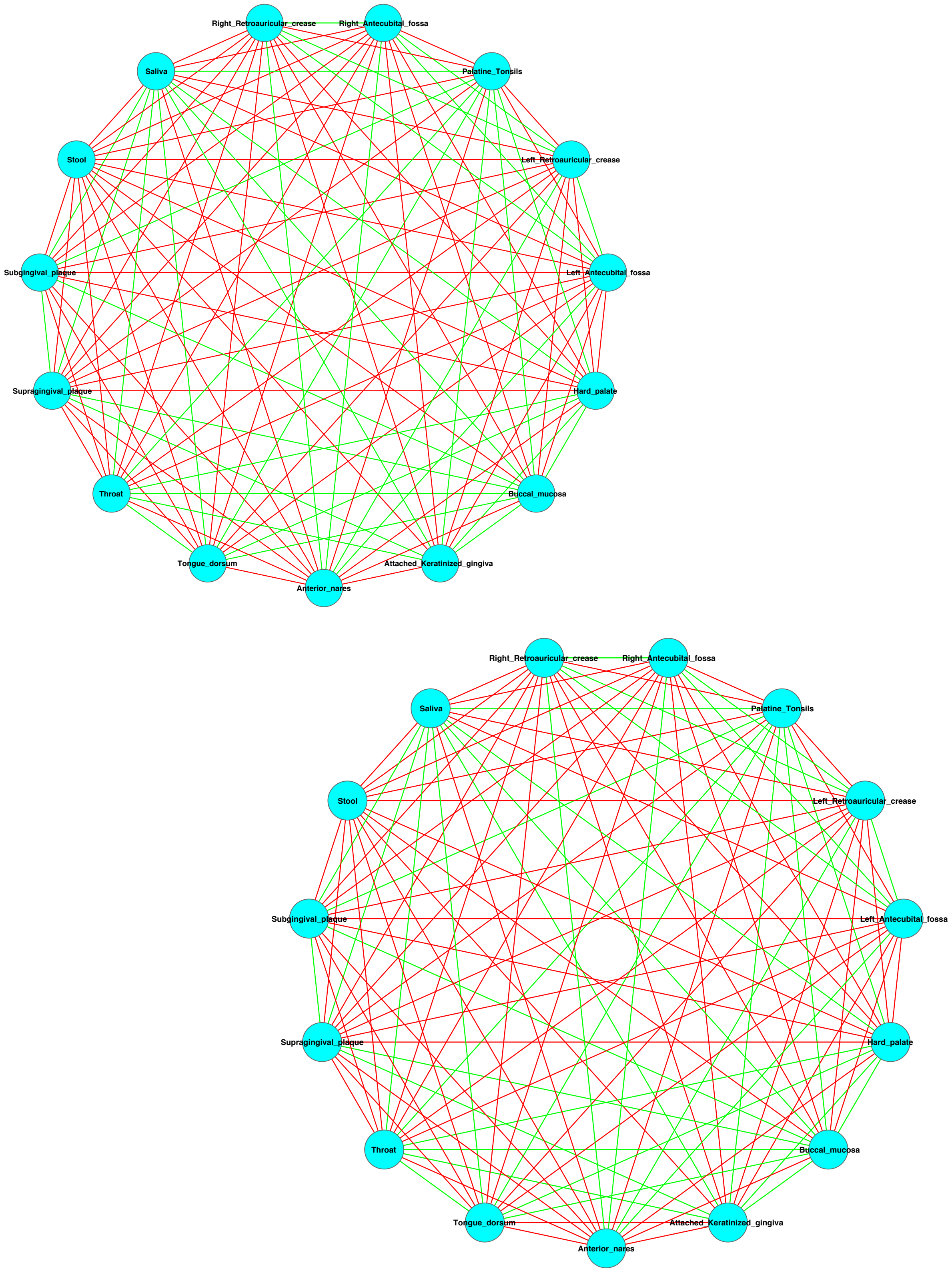

Fig S4. The site heterogeneity network (SHN) for the male (the top) and female (bottom) microbiomes across body sites: both the networks are virtually the same. In the main manuscript, each network is broken into positive and negative networks.

Part II: Tables S7-S8 & Figures S5-S6 (Computed from Mandar *et al*. (2015) complementary seminovaginal microbiomes datasets)

Table 7A. The specificity diversity (SD) permutation (SDP) tests for the species category of unique species (US) in the former case, computed from Mandar et al. (2015) complementary seminovaginal microbiomes datasets

Treatments	Order	Former case	Latter case	Delta	Lower (95%)	Upper (95%)	*p*-Value
CM vs. CNA	*q=0*	53.000	0.000	53.000	-3.381	3.271	0.000
	q=1	49.144	0.000	49.144	-10.228	9.772	0.000
	q=2	45.928	0.000	45.928	-12.356	11.755	0.000
	q=3	43.196	0.000	43.196	-13.231	12.549	0.000
	q=4	40.863	0.000	40.863	-13.634	12.899	0.000
CM vs. CNB	*q=0*	70.000	0.000	70.000	-6.246	6.206	0.000
	q=1	59.921	0.000	59.921	-10.542	10.726	0.000
	q=2	53.522	0.000	53.522	-11.868	12.075	0.000
	q=3	48.967	0.000	48.967	-12.218	12.390	0.000
	q=4	45.566	0.000	45.566	-12.286	12.410	0.000
CNA vs. CNB	*q=0*	46.000	0.000	46.000	-39.412	40.528	0.000
	q=1	40.087	0.000	40.087	-38.011	39.005	0.000
	q=2	35.280	0.000	35.280	-35.829	36.722	0.000
	q=3	31.749	0.000	31.749	-33.700	34.522	0.000
	q=4	29.199	0.000	29.199	-31.878	32.649	0.003

Table 7B. The specificity diversity (SD) permutation (SDP) tests for the species category of unique species (US) in latter case, computed from Mandar et al. (2015) complementary seminovaginal microbiomes datasets

Treatments	Order	Former case	Latter case	Delta	Lower (95%)	Upper (95%)	*p*-Value
CM vs. CNA	*q=0*	0.000	10.000	-10.000	-8.687	9.183	0.000
	q=1	0.000	9.617	-9.617	-9.301	9.780	0.000
	q=2	0.000	9.308	-9.308	-9.393	9.856	0.000
	q=3	0.000	9.050	-9.050	-9.226	9.670	0.000
	q=4	0.000	8.832	-8.832	-9.013	9.439	0.000
CM vs. CNB	*q=0*	0.000	6.000	-6.000	-5.426	5.210	0.000
	q=1	0.000	5.586	-5.586	-5.077	4.924	0.000
	q=2	0.000	5.261	-5.261	-4.783	4.661	0.000
	q=3	0.000	5.011	-5.011	-4.552	4.449	0.000
	q=4	0.000	4.817	-4.817	-4.382	4.290	0.000
CNA vs. CNB	*q=0*	0.000	25.000	-25.000	-15.753	15.075	0.000
	q=1	0.000	19.654	-19.654	-14.545	13.975	0.000
	q=2	0.000	14.826	-14.826	-13.084	12.590	0.019
	q=3	0.000	11.680	-11.680	-11.941	11.490	0.043
	q=4	0.000	9.913	-9.913	-11.101	10.679	0.075

Table 7C. The specificity diversity (SD) permutation (SDP) tests for the species category of enriched species (ES) in the former case, computed from Mandar et al. (2015) complementary seminovaginal microbiomes datasets

Treatments	Order	Former case	Latter case	Delta	Lower (95%)	Upper (95%)	*p*-Value
CM vs. CNA	***q=0***	126.000	126.000	0.000	-0.934	0.892	0.166
	q=1	116.690	49.865	66.824	-13.126	12.442	0.000
	q=2	109.608	31.603	78.004	-18.848	17.813	0.000
	q=3	104.242	24.294	79.948	-22.423	21.165	0.000
	q=4	100.141	20.404	79.737	-24.989	23.576	0.000
CM vs. CNB	***q=0***	107.000	107.000	0.000	-0.615	0.599	0.070
	q=1	99.960	44.933	55.027	-13.110	13.119	0.000
	q=2	94.390	31.577	62.812	-18.188	18.194	0.000
	q=3	90.049	26.680	63.369	-20.769	20.792	0.000
	q=4	86.662	24.191	62.471	-22.288	22.339	0.000
CNA vs. CNB	***q=0***	0.000	0.000	0.000	0.000	0.000	0.000
	q=1	0.000	0.000	0.000	0.000	0.000	0.000
	q=2	0.000	0.000	0.000	0.000	0.000	0.000
	q=3	0.000	0.000	0.000	0.000	0.000	0.000
	q=4	0.000	0.000	0.000	0.000	0.000	0.000

Table 7D. The specificity diversity (SD) permutation (SDP) tests for the species category of enriched species (ES) in the latter case, computed from Mandar et al. (2015) complementary seminovaginal microbiomes datasets

Treatments	Order	Former case	Latter case	Delta	Lower (95%)	Upper (95%)	*p*-Value
CM vs. CNA	***q=0***	5.000	5.000	0.000	-0.259	0.253	0.017
	q=1	3.334	4.590	-1.256	-1.067	1.131	0.018
	q=2	3.118	4.369	-1.251	-1.111	1.159	0.024
	q=3	3.049	4.243	-1.193	-1.074	1.113	0.025
	q=4	3.011	4.163	-1.152	-1.035	1.068	0.024
CM vs. CNB	***q=0***	3.000	3.000	0.000	-0.140	0.138	0.005
	q=1	1.406	2.786	-1.379	-1.530	1.431	0.027
	q=2	1.205	2.623	-1.419	-1.808	1.695	0.063
	q=3	1.157	2.506	-1.349	-1.825	1.712	0.086
	q=4	1.138	2.421	-1.282	-1.794	1.685	0.102
CNA vs. CNB	***q=0***	2.000	2.000	0.000	-0.088	0.088	0.002
	q=1	1.147	1.919	-0.772	-1.166	1.177	0.219
	q=2	1.063	1.849	-0.785	-1.308	1.319	0.362
	q=3	1.048	1.792	-0.744	-1.308	1.319	0.413
	q=4	1.042	1.748	-0.706	-1.286	1.296	0.433

Table 7E. The specificity diversity (SD) permutation (SDP) tests for the species category of "all species with significant differences in specificity", computed from Mandar et al. (2015) complementary seminovaginal microbiomes datasets

Treatments	Order	Former case	Latter case	Delta	Lower (95%)	Upper (95%)	*p*-Value
CM vs. CNA	***q=0***	184.000	141.000	43.000	-9.110	9.448	0.000
	q=1	165.171	38.127	127.044	-28.556	27.449	0.000
	q=2	152.136	19.536	132.600	-41.030	39.098	0.000
	q=3	142.453	14.516	127.937	-49.362	46.942	0.000
	q=4	135.212	12.581	122.631	-54.929	52.220	0.000
CM vs. CNB	***q=0***	180.000	116.000	64.000	-7.485	7.211	0.000
	q=1	158.241	35.684	122.557	-27.249	27.504	0.000
	q=2	145.255	17.969	127.286	-36.760	37.091	0.000
	q=3	136.132	12.417	123.715	-41.595	41.906	0.000
	q=4	129.420	10.218	119.202	-44.316	44.575	0.000
CNA vs. CNB	***q=0***	48.000	27.000	21.000	-40.398	40.836	0.345
	q=1	41.191	19.082	22.109	-39.901	39.927	0.343
	q=2	36.249	13.666	22.583	-37.829	37.516	0.297
	q=3	32.619	10.943	21.676	-34.866	34.399	0.267
	q=4	29.979	9.565	20.414	-31.909	31.397	0.246

Table 7F. The specificity diversity (SD) permutation (SDP) tests for the species category of "all species (with or without specificity differences)", computed from Mandar et al. (2015) complementary seminovaginal microbiomes

Treatments	Order	Former case	Latter case	Delta	Lower (95%)	Upper (95%)	*p*-Value
CM vs. CNA	***q=0***	259.000	216.000	43.000	-15.556	16.004	0.000
	q=1	206.767	78.455	128.312	-43.355	42.943	0.000
	q=2	182.362	42.905	139.457	-54.092	52.697	0.000
	q=3	167.138	31.240	135.898	-61.235	59.127	0.000
	q=4	156.733	26.449	130.285	-65.848	63.298	0.000
CM vs. CNB	***q=0***	259.000	195.000	64.000	-10.388	9.952	0.000
	q=1	209.717	71.495	138.223	-38.965	39.485	0.000
	q=2	184.556	43.258	141.297	-51.486	52.242	0.000
	q=3	168.882	33.481	135.401	-57.506	58.282	0.000
	q=4	158.254	28.988	129.267	-60.174	60.903	0.000
CNA vs. CNB	***q=0***	216.000	195.000	21.000	-59.059	59.815	0.487
	q=1	145.124	121.966	23.158	-73.299	74.830	0.603
	q=2	108.678	93.661	15.017	-65.711	67.269	0.689
	q=3	87.517	79.538	7.979	-58.384	59.787	0.807
	q=4	74.717	71.287	3.430	-53.071	54.318	0.921

Table S8. The numbers of species in different species categories groups, computed from Mandar *et al*. (2015) complementary seminovaginal microbiomes datasets

Treatment	With significant difference				Total of Significantly Different Species	Total of Insignificantly Different Species	Total Species	Shared Species from SSA
	US in Former	US in Latter	ES in Former	ES in Latter				
CM (Semen) *vs*. CNA (Vaginal before sex)	53	10	126	5	194	75	269	206** (77%)
CM (Semen) *vs*. CNB (Vaginal after sex)	70	6	107	3	186	79	265	189** (71%)
CNA (Vaginal before sex *vs*. CNB (Vaginal after sex)	46	25	0	2	73	168	241	170* (71%)

* There are significant fewer shared species between two groups per the SSA (shared species analysis) with reads-level permutation tests (Ma *et al*. 2019).

** There are significant fewer shared species between two groups per the SSA (shared species analysis) with reads-level permutation tests and sample-permutation tests (Ma *et al*. 2019).

Table S10. Specificity diversity permutation (SDP) tests for all negative-control treatments of gut microbiome of men and women, respectively **(No SD difference is detected in any tests below)**

**Datasets**	**Diversity Order**	**Data A**	**Data B**	**Delta**	**Lower Limit (95%)**	**Upper Limit (95%)**	***P*-value**
**Male *vs*. Male**							
Duplicated Treatment for Male	*q*=0	5733.000	5873.000	-140.000	-514.705	597.187	0.641
	q=1	3375.358	3522.216	-146.858	-565.251	601.576	0.619
	q=2	2371.665	2523.284	-151.619	-500.847	517.640	0.558
	q=3	1865.467	2031.088	-165.620	-429.842	439.005	0.464
Half-Divided Treatment for Male	*q*=0	6510.000	6417.000	93.000	-275.691	267.205	0.486
	q=1	3799.916	3544.514	255.402	-448.841	445.447	0.256
	q=2	2704.704	2440.357	264.348	-399.369	394.683	0.190
	q=3	2168.186	1928.941	239.245	-340.320	334.533	0.175
**Female *vs*. Female**							
Duplicated Treatment for Female	*q*=0	5870.000	5711.000	159.000	-682.442	658.240	0.649
	q=1	3607.650	3378.801	228.849	-611.282	576.830	0.474
	q=2	2577.764	2322.588	255.176	-549.827	517.581	0.360
	q=3	2043.512	1787.898	255.614	-495.936	467.786	0.309
Half-Divided Treatment for Female	*q*=0	6422.000	6638.000	-216.000	-336.341	333.795	0.207
	q=1	3476.763	3827.929	-351.167	-472.413	459.047	0.136
	q=2	2371.523	2705.072	-333.549	-428.573	413.431	0.118
	q=3	1857.933	2166.696	-308.763	-381.855	367.115	0.096

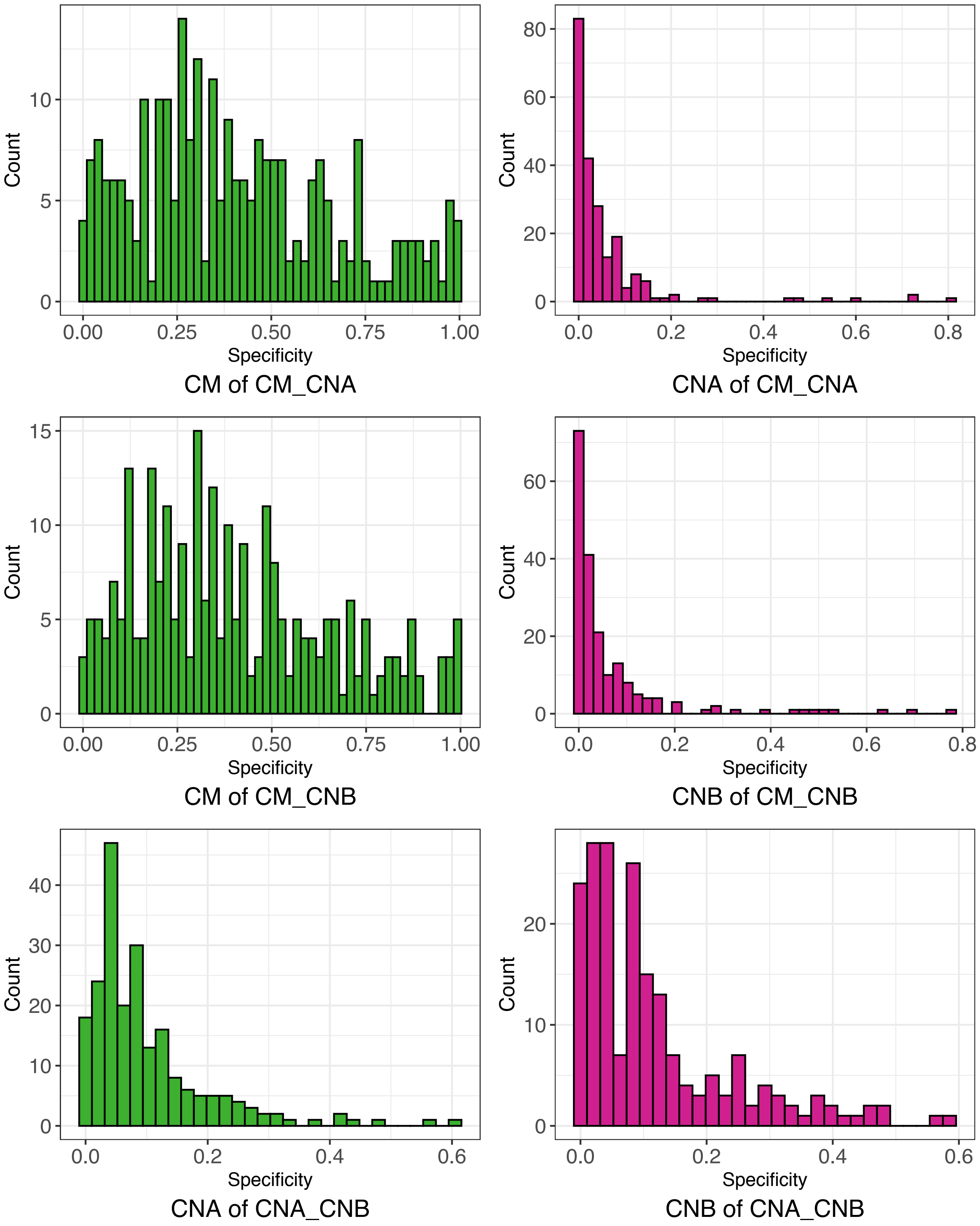

Fig S5. Histograms of the species specificity (SS) of the seminovaginal microbiome (SVM) comparsions: CM (semen) vs. CNA (vaginal before sex), CM (semem) vs. CNB (vaginal after sex), and CNA vs. CNB.

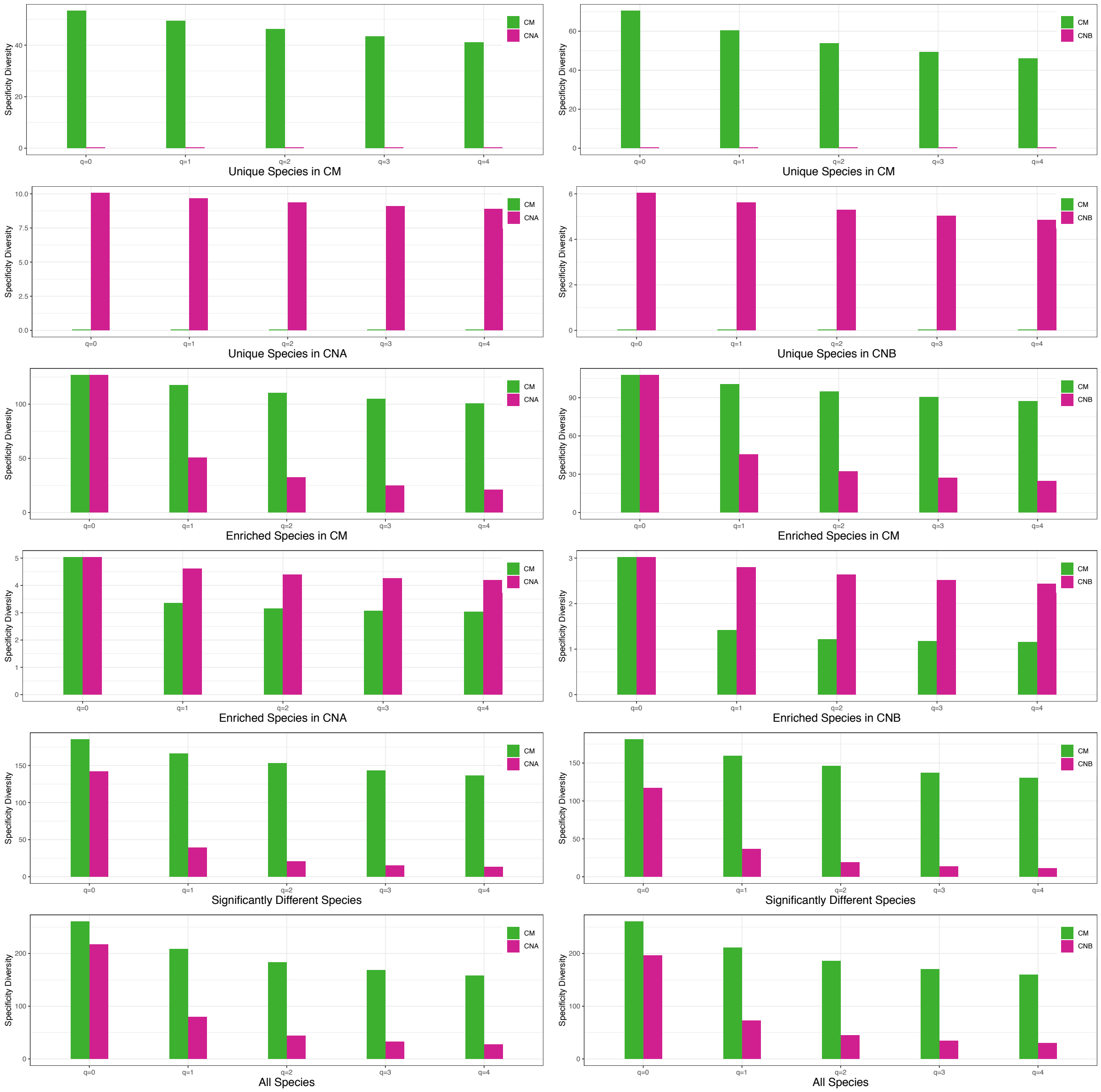

Fig S6. The SDP (specificity diversity permutation) tests for the SVM (seminovaginal microbiome) comparsions: the left for the CM (semen) vs. CNA (vaginal before sex), CM (semen) vs. CNB (vaginal after sex).
